# Multilayered Curcumin-Loaded Hydrogel Microcarriers with Antimicrobial Function

**DOI:** 10.3390/molecules27041415

**Published:** 2022-02-19

**Authors:** Weronika Szczęsna, Marta Tsirigotis-Maniecka, Łukasz Lamch, Lilianna Szyk-Warszyńska, Ewa Zboińska, Piotr Warszyński, Kazimiera A. Wilk

**Affiliations:** 1 Department of Engineering and Technology of Chemical Processes, Faculty of Chemistry, Wrocław University of Science and Technology, 50-370 Wrocław, Poland; weronika.szczesna@pwr.edu.pl (W.S.); marta.tsirigotis@pwr.edu.pl (M.T.-M.); lukasz.lamch@pwr.edu.pl (Ł.L.); 2 Jerzy Haber Institute of Catalysis and Surface Chemistry, Polish Academy of Sciences, 30-239 Kraków, Poland; lilianna.szyk-warszynska@ikifp.edu.pl; 3 Department of Organic and Medicinal Chemistry, Faculty of Chemistry, Wrocław University of Science and Technology, 50-370 Wrocław, Poland; ewa.zboinska@pwr.edu.pl

**Keywords:** multifunctional microcarriers, curcumin, decorated poly(acrylic acid), layer-by-layer (LbL) methodology, antimicrobial function

## Abstract

The design of multifunctional microcarriers has attracted significant attention because they combine various functions within a single system. In this study, we developed a set of multilayered hydrogel microcarriers, which were first loaded with chemotherapeutic curcumin (CUR), then, using the layer-by-layer (LbL) technique, coated through a polyelectrolyte shell consisting of chitosan (CHIT) or poly(allylamine hydrochloride) (PAH). As an outer layer with antimicrobial function, newly synthesised alkylene quaternary ammonium salt functionalised polyelectrolytes (A-QAS-PEs) were applied. For this purpose, poly(acrylic acid) (PAA) was decorated with different hydrophobic side chains (*n*-hexane and *n*-dodecane side entities) and different degrees of substitution (m) of quaternary ammonium groups (abbreviated as PAA-C(O)O-(CH_2_)_n_-N^+^(CH_3_)_3_(m); n = 6, 12; m = 8–14%). The grafting approach of PAA with the alkylene quaternary ammonium salt moiety was performed under mild reaction conditions using Steglich esterification followed by quaternisation. The structure of antimicrobial decorated PAA was confirmed by ^1^H NMR and FTIR, and the mean diameter of all multifunctional microparticles was characterised by SEM. The viscoelastic properties of the functional layers were studied using quartz crystal microbalance with a dissipation (QCM-D). The release of CUR from the microcarriers was described using a hybrid model, i.e., a combination of first-order kinetics and the Korsmeyer-Peppas model. The antimicrobial activity of functionalised PAA and multilayered CUR-loaded hydrogel microcarriers with quaternary ammonium function was assessed against *Staphylococcus aureus* and *Serratia marcescens* by the agar diffusion assay method. Only a limited inhibition zone of PAA was observed, but in the case of both antimicrobial decorated PAA and the corresponding multilayered nanocarriers, the inhibitory activity increase was achieved against both strains of bacteria.

## 1. Introduction

In recent years, bacterial infections, especially those that occur after antibiotic treatment, have become a major problem worldwide as microbes are becoming increasingly resistant to antibiotics and other antimicrobial agents [1,2]. Thus, new biocidal strategies that do not require antibiotics should be developed and employed to treat infections caused by pathogenic microbes. One of the promising approaches for developing effective antimicrobial systems appears to be the surface functionalisation of carriers for drug delivery [3,4]. Among these strategies, the layer-by-layer (LbL) technique is a versatile methodology to construct functional surfaces by alternate deposition of polycation and polyanion [5]. The LbL approach can be used to control the buildup of multilayer coatings of different architectures and functions on the nanometer scale [6,7]. The simplicity of LbL film formation enables one to design a variety of custom-designed materials with assumed antimicrobial features. The design and fabrication of multifunctional hydrogel microparticles have recently attracted great interest because of their ability to put together various functions within a single entity [8]. The formulations used typically contain carriers at a relatively high concentration, and the Brownian motion to which they are subjected would not, therefore, adversely affect the efficiency of the payload delivery. Carriers functionalization can increase delivery efficacy due to prolonged and/or selective interactions at the site of action, resulting in more precise targeting. The design and provision of various functionalities to microcarriers expands their application potential. In addition to their primary role, i.e., the ability to deliver a biologically active substance to the site of action in the body, they gain an additional feature, i.e., selective antimicrobial activity, which allows multifaceted therapy in a short time. For nanocarriers, the most common surface functionalization comprises covalent bonding with appropriate molecules enabling selective interaction with the targeting receptor. On the other hand, the surface of larger colloidal carrier systems (e.g., microparticles) is functionalized by attaching an appropriate chemical function such as antimicrobial, antifouling, antiadhesive or biofilm preventive actions [3,9,10,11]. Furthermore, multifunctional microcarriers can be easily coated with various functional films that incorporate additional functionality of the whole microsystem. In this way, we can design multifunctional microparticles in which the payload and the shell can have different roles. Microparticles that have multiple components and features can be easily adjusted to fulfil the requirements of desirable applications. In the fabrication of such microcarriers, the choice of appropriate material as a building block is crucial [12]. Biocompatible natural polymers such as sodium alginate (ALG) and chitosan (CHIT), as well as synthetic polymers such as poly(allylamine hydrochloride) (PAH) and poly(acrylic acid) (PAA), are considered valuable building blocks for a broad range of drug delivery systems (DDS) that successfully provide the ability of the payload-controlled release at the site of action. 

Several natural and synthetic polyelectrolytes (PEs) with various functional moieties have been used as microparticle coatings. Polymers bearing positively charged groups are potential antimicrobial agents that can adhere to the negatively charged bacterial surface, causing structural disruption of the cell membrane. Moreover, numerous cationic PEs are generally recognized as safe (GRAS), especially polysaccharides and their derivatives; therefore, they have emerged as promising compounds because of their antibacterial properties and biocompatibility [4,13]. Among polyelectrolytes with various cationic moieties, quaternary ammonium compounds offer high efficacy in preventing the growth of bacteria [14,15,16,17]. The key factor providing the antimicrobial properties of the polymer is the degree of substitution of a functional group or alkyl side chain length. Therefore, the design of functionalised polyelectrolytes with the appropriate hydrophobic segments and cationic moieties is crucial for obtaining high antibacterial efficiency. Multifunctional PEs can be synthesised using various approaches, such as direct copolymerisation or polycondensation, and multistep processes involving the preparation of the polymer backbone and its functionalisation by coupling with various chemical moieties for quaternisation, hydrolysis, and other functions [18,19,20,21]. In general, polyelectrolyte functionalisation, especially through decoration with short side groups, leads to products with random structures [18]. This strategy often involves steps under mild conditions, such as Steglich esterification or amidation, to avoid degradation of the backbone [22,23]. Polyelectrolytes with hydrophilic/hydrophobic side chains of various lengths with a suitable active moiety (e.g., ligand or antimicrobial group) attached to one of their ends are synthesised from suitable asymmetric precursors, such as α,ω-substituted olefins or poly(ethylene oxides) [24]. The use of the above-mentioned derivatives avoids undesirable processes such as crosslinking or the formation of water-insoluble by-products (polymers with insufficient grafting). In addition, further end-group reactions should be selective to avoid unnecessary reactions within the polymer backbone.

The literature reports show that nano and microcapsules with functional coatings applied by the LbL technique are worth considering for their application in antimicrobial therapy. The antibacterial properties of such structures can be adjusted by controlling the chemical composition and architecture of the capsules. For example, Ivanova et al. [4] designed polymer nanoparticles coated with cationic aminocellulose and anionic glycosaminoglycan—hyaluronic acid. Such nanosized materials showed strong antibacterial efficiency against both the Gram-positive bacteria *S. aureus* and the Gram-negative bacteria *E. coli* without affecting the viability of human fibroblast cells. In other work, researchers prepared microcapsules composed of alginate and polycyclodextrin that contained quaternary ammonium moieties as antimicrobial agents. The fabricated microparticles demonstrated an inhibitory effect against *E. coli* and *S. aureus* growth [25]. Therefore, the functionalisation of drug carriers with antimicrobial coatings using the LbL approach has great potential in various therapies.

Curcumin (CUR) is a natural polyphenolic compound with promising health benefits due to its valuable biological activities, such as antioxidant [26], anticarcinogenic [27], antidiabetic [28], antiproliferative [29], and anti-inflammatory [30] activities. CUR has attracted widespread attention in the treatment or prevention of numerous diseases such as cancer [31], and liver [32], lung [33], autoimmune [34], neurological [34] or cardiovascular [35] diseases. For this reason, several studies have reported the encapsulation of CUR into microparticles for potential therapeutic applications [36]. Our group has recently prepared and characterised hydrogel micro and macroparticles based on ALG, CHIT, and gelatin to deliver curcumin to colon cancer cells [27]. As a continuation of our studies on rational drug delivery [37,38], we designed, fabricated, and characterised multilayered hydrogel microcarriers comprising the CUR-loaded core and custom-designed LbL coatings, the latter formed by CHIT or PAH and, as the outer antimicrobial layer, functionalized poly(acrylic acid) alkylene quaternary ammonium salt. Our system comprised two completely independent functions: chemotherapeutic and antimicrobial. For the first purpose, we used curcumin as the payload for sustained release, and for the second we used quaternary ammonium groups covalently attached to the polyelectrolyte, constituting the external layer of the microparticles. Such multifunctionality may be particularly useful for combating multidrug-resistant bacteria but is not limited to this purpose. It should be noted that both quaternary ammonium compounds, as well as curcumin, have been widely studied for their antimicrobial and chemotherapeutic activity and safety for higher organisms. Moreover, the use of very mild polyelectrolytes avoids harmful effects connected with high activity of low-molecular-weight surfactants at biological membranes. Therefore, the above-mentioned multifunctional microcarriers were composed of ALG, used as a core material, CHIT or PAH selected as the first layer, and a newly synthesised antimicrobial decorated poly(acrylic acid), deposited as the outer layer to provide the assumed functional coating. The morphology and composition of all fabricated microparticles were studied with a scanning electron microscope (SEM) and Fourier transform infrared (FTIR) spectroscopy, respectively. We evaluated the thickness, mass, and viscoelastic properties of multilayer films during their build-up using a quartz crystal microbalance with a dissipation (QCM-D) technique. In addition, the CUR kinetic release profiles and mechanisms were thoroughly analysed. Finally, we evaluated the influence of the coating characteristics (i.e., thickness, type of layer, polyelectrolyte functionalization) on the antimicrobial activity of the prepared microcarriers against Gram-positive (*Staphylococcus aureus*) and Gram-negative (*Serratia marcescens*) bacterial strains. The beneficial physicochemical and biological properties acquired of the fabricated microparticles could allow the design of systems to deliver pharmaceuticals (e.g., CUR) by oral or intravesical administration in various methods of treatment, including antimicrobial and cancer therapies [39,40]. 

## 2. Results and Discussion

Microcarriers with multilayer coatings built of modified polyelectrolytes of various functions constitute a promising group of DDS [41,42]. In addition, rationally engineered polyelectrolytes, such as alkylene quaternary ammonium salt functionalised polyelectrolytes (A-QAS-PEs), can be used as building blocks for multilayered microparticles. Using such polymers to impart new carrier properties via the LbL approach is a well-proven strategy that allows one to form a variety of tailor-made coatings exhibiting antimicrobial activity [43]. Thus, A-QAS-PEs may extend the conventional group of decorated PEs, opening new frontiers for their fabrication and characterising their unique properties.

### 2.1. Design, Grafting, and Characterisation of Poly(acrylic acid) Containing Alkylene Quaternary Ammonium Groupings

Antimicrobial actions of multilayer coatings can be achieved either by using PEs with intrinsic antimicrobial function [44] or by embedding in the film structure active components as metallic nanoparticles [45] or drug-containing nanocapsules [46,47]. Functionalized PEs that show antimicrobial activities may be divided into three main groups: polymeric biocides, biocidal polymers, and biocide-releasing polymers [48]. For the preparation of nano and microcarriers, showing intrinsic antimicrobial function, the second group of macromolecules, i.e., biocidal polymers, is particularly useful. The aforementioned function of the polymers is connected with the fact that, generally, microbial cells carry a negative net charge at the surface due to their membrane proteins, teichoic acids of Gram-positive bacteria, and negatively charged phospholipids at the outer membrane of Gram-negative bacteria. In this way, polycations (e.g., CHIT), if they have a proportionate amphiphilic character, can disrupt the outer and the cytoplasmic membrane and cause cell lysis, resulting in cell death. Numerous polymeric biocides possess cationic groups positioned along the polymer backbone, but there is also a very effective class of antimicrobial polymers with a biocidal end of the main or backside chain. Chemical moieties, mainly possessing a quaternary ammonium salt and/or an oxazoline group, may play an antimicrobial role at very low concentrations, e.g., one end group per polymer. Coupling the mentioned group with a long alkyl chain may provide additional control of the whole molecule activity in the range of three orders of magnitude by the group distal to the biocide: a so-called satellite group [49]. The strategy for attaching a long alkyl chain and a labile moiety as the linking groups between the quaternary ammonium salt and the polymer backbone is a novel synthesis approach for biocidal polymeric materials. 

**Scheme 1:**
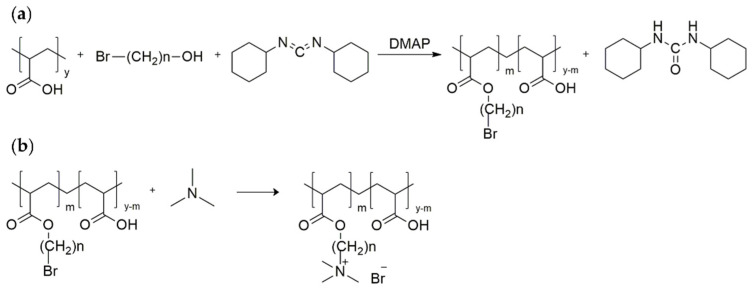
(**a**) Synthetic route of poly(acrylic acid) with a bromine-terminated side alkyl chain (hexyl or dodecyl) obtained via Steglich esterification; (**b**) synthesis of poly(acrylic acid) containing alkylene quaternary ammonium groupings by quaternisation with trimethylamine.

An alkylene quaternary ammonium salt decorated PAA was prepared in a two-step reaction (Scheme 1). In the first step, poly(acrylic acid) modified with a bromine-terminated hydrophobic chain was synthesised under mild conditions by Steglich esterification with N,N-dicyclohexylcarbodiimide (DCC) as a coupling agent and 4-dimethylaminopyridine (DMAP) as a catalyst [22,23]. Subsequently, bromine-terminated poly (acrylic acid) was converted to a quaternary ammonium salt by quaternisation with trimethylamine. Therefore, we decorated poly(acrylic acid) with various hydrophobic chains (*n*-hexane and *n*-dodecane side entities) and different degrees of substitution (m) for quaternary ammonium groups (abbreviated as PAA-C(O)O-(CH_2_)_n_-N^+^(CH_3_)_3_(m); n = 6, 12; m = 8, 10, 14%; Table 1). 

To avoid the undesirable reaction of trimethylamine with acid groups of poly(acrylic acid), the intermediate was neutralised after Steglich esterification with alcoholic sodium hydroxide solution. Direct quaternisation with a 10-fold excess of trimethylamine was performed at 4 °C in a tightly closed vessel to avoid evaporation of the volatile reagent. The above-mentioned synthesis allowed us to obtain the desired products under mild conditions, using simple purification methods (dialysis, freeze-drying). It should be emphasised that the synthetic routes may constitute general repetitive methods of fabrication of numerous antimicrobial polyelectrolytes.

**Figure 1:**
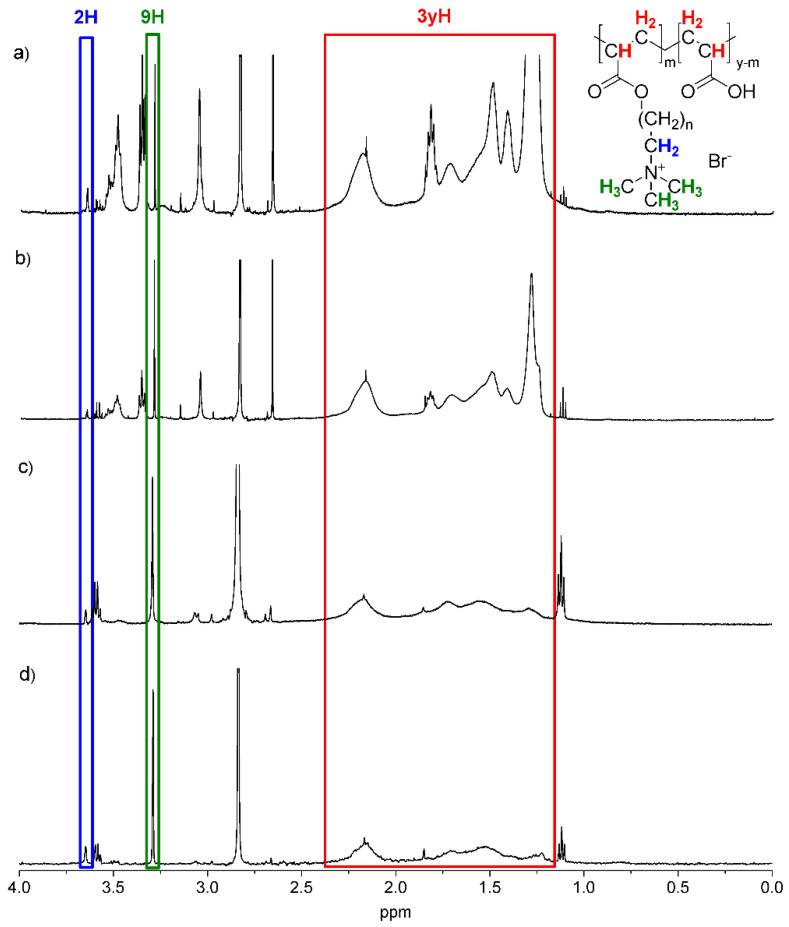
^1^H-NMR spectra of PAA-C(O)O-(CH_2_)_12_-N^+^(CH_3_)_3_-14% (**a**), PAA-C(O)O-(CH_2_)_12_-N^+^(CH_3_)_3_-8% (**b**), PAA-C(O)O-(CH_2_)_6_-N^+^(CH_3_)_3_-10% (**c**) and PAA-C(O)O-(CH_2_)_6_-N^+^(CH_3_)_3_-8% (**d**) with marked signals attributed to the identified protons. Blue line means 2H, green line means 9H, red line means 3yH.

**Figure 2:**
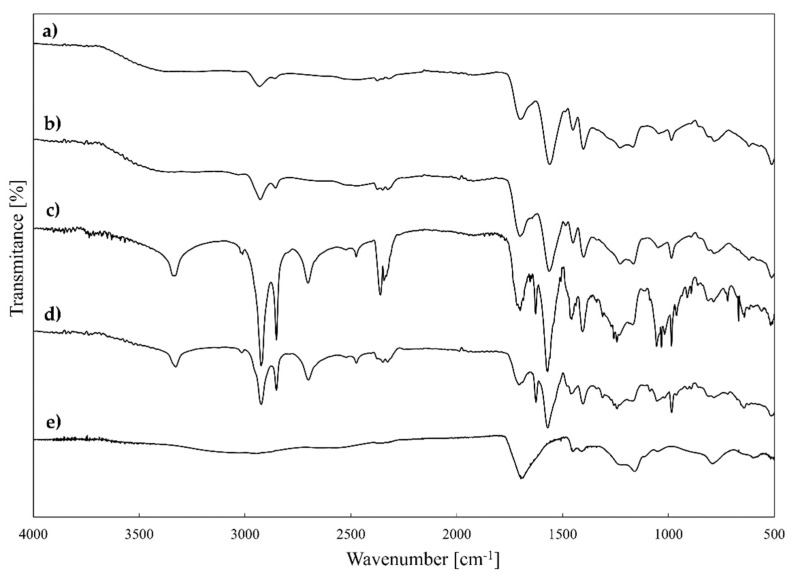
FTIR spectra of PAA-C(O)O-(CH_2_)_6_-N^+^(CH_3_)_3_-8% (a), PAA-C(O)O(CH_2_)_6_N^+^(CH_3_)_3_-10% (b), PAAC(O)O(CH_2_)_12_N^+^(CH_3_)_3_-8% (c), PAAC(O)O(CH_2_)_12_N^+^(CH_3_)_3_-14% (d) and PAA (e).

The molecular structures of all antimicrobial functionalised PAA derivatives **1**–**4** were determined by ^1^H NMR and FTIR techniques (Figure 1 and Figure 2, respectively). In the ^1^H NMR spectra (for PAA modified with hexyl: PAA-C(O)O-(CH_2_)_6_-N^+^(CH_3_)_3_-8% and PAA-C(O)O-(CH_2_)_6_-N^+^(CH_3_)_3_-10%, or dodecyl: PAA-C(O)O-(CH_2_)_12_-N^+^(CH_3_)_3_-8% and PAA-C(O)O-(CH_2_)_12_-N^+^(CH_3_)_3_-14%, the alkyl chain ended with a quaternary ammonium moiety; see Figure 1), and a sharp signal was assigned to three methyl groups attached directly to the quaternary nitrogen atom (chemical shift 3.3 ppm). Moreover, a weaker signal (4.5 times lower by integration intensity, chemical shift 3.6 ppm) was identified and attributed to the methylene moiety directly bonded to the quaternary ammonium moiety. Signals attributed to the repeating methylene and methine groups in the polyelectrolyte backbone, as well as repeating methylene moieties in the side chains, appeared as broad signals in the region of 1.1–2.4 ppm [22]. It was impossible to assign the signals directly due to their overlap.

The values of the grafting ratio and degree of substitution (percentage of grafting) were estimated from ^1^H NMR spectra taking into account integrated signal intensities at 3.3 ppm and in the range of 1.1–2.4 ppm according to the protocol described by Wawrzynczyk et al. [24]. The values of the grafting ratio (i.e., number of units (mers) in the PE backbone per unit grafted with the cationic group) were 11.8 (PAA-C(O)O-(CH_2_)_6_-N^+^(CH_3_)_3_-8%), 9.7 (PAA-C(O)O-(CH_2_)_6_-N^+^(CH_3_)_3_-10%), 11.2 (PAA-C(O)O-(CH_2_)_12_-N^+^(CH_3_)_3_-8%) and 7.4 (PAA-C(O)O-(CH_2_)_12_-N^+^(CH_3_)_3_-14%). The corresponding grafting percentages were found to be 8% (PAA-C(O)O-(CH_2_)_6_-N^+^(CH_3_)_3_-8%), 10% (PAA-C(O)O-(CH_2_)_6_-N^+^(CH_3_)_3_-10%), 8% (PAA-C(O)O-(CH_2_)_12_-N^+^(CH_3_)_3_-8%) and 14% (PAA-C(O)O-(CH_2_)_12_-N^+^(CH_3_)_3_-14%). The assumed values of grafting ratios (8–14%) were chosen to obtain negatively charged polymers. It has been proven that polyanions with less than 15% of mers bearing cationic groups maintained their anionic character [50].

FTIR studies of synthesised A-QAS-PEs (products **1**–**4**, Table 1) showed the presence of characteristic bands assigned to O-H, C-H, C=O, C-O-C, and C-N bonds that confirm their presence in functionalised polyelectrolytes. The presence of both carboxylate and ester groups was confirmed by signals (attributed to carbonyl stretching vibrations) at around 1643–1666 cm^−1^ and around 1702–1708 cm^−1^, respectively. The signals around 1465–1478 cm^−1^ correspond to C-N bonds [51] in the quaternary ammonium moiety. However, they are partially overlapped with signals from alkyl C-H bonds (CH_3_ and CH_2_ groups).

### 2.2. Characterisation of the Obtained Multifunctional Microcarriers

**Figure 3:**
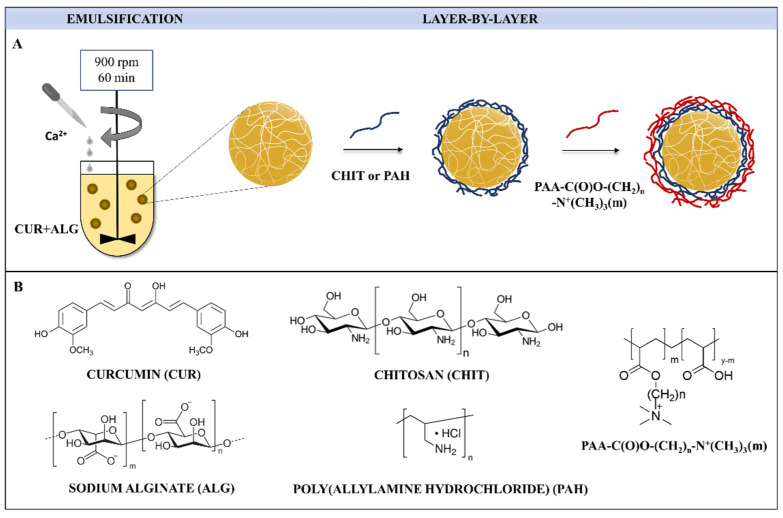
Fabrication process of bilayer CUR-loaded microparticles coated with alkylene quaternary ammonium salt grafted poly(acrylic acid) (denoted as PAA-C(O)O-(CH_2_)_n_-N^+^(CH_3_)_3_(m); n = 6, 12; m = 8, 10, 14%; Table 1) as the outer layer (**A**); structures of microparticle components (**B**).

Strategies to construct properly designed microcarriers for multiple applications are guided by various factors: (i) appropriate physicochemical features: shape, size, stiffness, morphology, and surface charge; (ii) type of building blocks, which should be of a biocompatible matter; (iii) appropriate chemical and colloidal stability; (iv) efficacious payload encapsulation; (v) ease of surface functionalisation [27,37,38]. Taking these factors into account, the synthetic route of microcapsules consisted of two major steps and, thus, integrated an emulsion method [27] to obtain alginate-based microspheres loaded with CUR, and the LbL technique [37] to form bilayer microcapsules coated with CHIT or PAH and antimicrobial decorated polyelectrolytes **1**–**4** (for details, see Table 1: PAA-C(O)O-(CH_2_)_n_-N^+^(CH_3_)_3_(m); n = 6, 12; m = 8, 10, 14%), as shown in Figure 3. Two microcapsule systems were formed, differing in the number and type of coatings. Based on our previous research, CHIT and PAH polymers were selected for the first coating layer for hydrogel microsystems [27,52]. Then, hydrogels were coated with **1**–**4** polymers as the second layer. The concentrations of the core polymer and the payload used were the same in all types of microparticles fabricated. Variations in the composition, abbreviations, and characteristics of the fabricated bilayer microcarriers are presented in Table 2.

**Figure 4:**
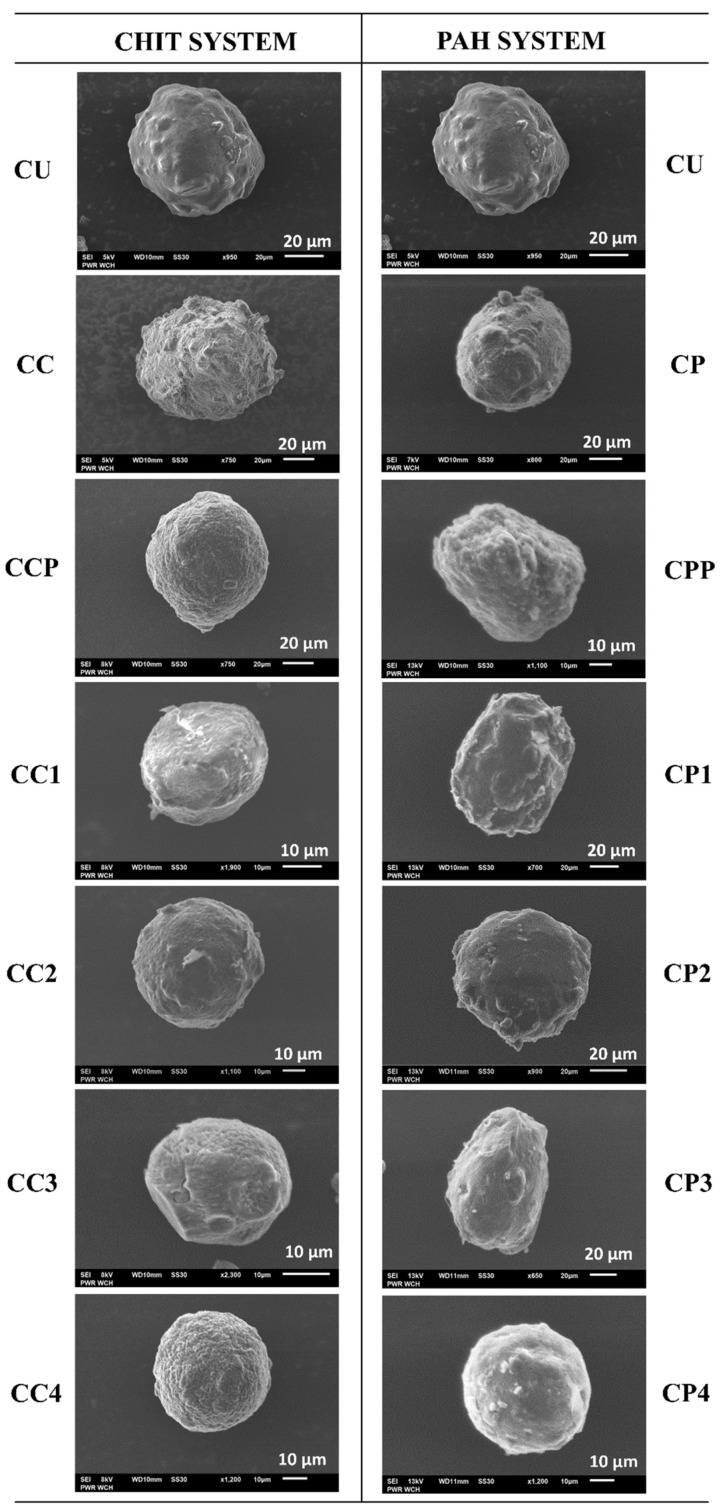
SEM images of CUR-loaded microparticles of CHIT and PAH systems (for details, see Table 2).

Investigations of the surface morphology and shape of the dried microparticles were carried out by SEM, and representative micrographs are shown in Figure 4. Detailed studies of the morphological structure of the carriers lead to conclusions concerning the changes in their surface caused by A-QAS-PEs. Figure 4 shows SEM pictures of an example of an average particle; however, the discussion applies to the entire population of microparticles of each type. Modifying the surface of the particles may change their mechanical properties and performance as carrier devices, i.e., cause the formation of large pores and cracks, or alter their original shape. Most of the microparticles were of similar spherical shape, and the polyelectrolyte coatings did not cause any significant changes in the shape of the microcapsules. However, there were differences in surface morphology. SEM images showed that the surface of microparticles functionalised with antimicrobial decorated PAA slightly differed depending on the type of polyelectrolyte **1**–**4** (see Table 1) used as the outer layer. In the case of the CHIT system microcarriers, the CC3 and CC4 surfaces were more porous and wrinkled than those of CC1 and CC2, probably due to the evaporation of a larger amount of water trapped within the outer layer of the microparticles. The external surfaces of microcapsules with PAH turned out to be similar to each other. Moreover, the CUs (for details see Table 2) had a smoother surface than the other microcarriers. Comparing all coated microparticles, it is clearly visible that the CPs (for details see Table 2) had a less rugged and irregular surface than other microcapsules. Thus, the PE shell appears to be the factor that affects the surface morphology. Similar findings have previously been observed for hydrogel particles coated with various polyelectrolyte shells, such as gelatine and chitosan [38]. 

The mean diameter values (MD) of all fabricated bilayer microparticles are shown in Table 2. Examination of the size of the microcarriers revealed some differences in MD between microspheres and coated microcapsules. The uncoated microparticles were characterised by a lower MD (45.9 ± 4.9 µm) than the coated ones. Furthermore, the MD of PAH-coated microcapsules (CP, CPP, CP1, CP2, CP3, and CP4) was higher than the MD of the CHIT-coated microcapsules (CC, CCP, CC1, CC2, CC3, and CC4), possibly due to the acidic conditions during the deposition of the chitosan layer. This resulted in the reduced repulsive interactions of alginate carboxyl groups and shrinkage of the alginate core. Further coating of the microparticles with either PAA or **1**–**4** alkylene quaternary ammonium salt functionalised poly(acrylic acid) did not change their size. The PdI values of the CU- and CHIT-coated microparticles indicate a moderate polydispersity of their size (0.100–0.400), whereas the PdI values of the PAH-coated microparticles appear to be much lower, and the population can be considered as monodisperse. PAH coated microparticles were of higher quality in terms of size distribution, which is very desirable in the formulation of a commercial product [53]. Our results suggest that the type of the first PE coating and the conditions of its deposition had the most significant effect on the size of the microcapsules. This outcome is consistent with a report that described the impact of different PE shells (chitosan, gelatin, poly(allylamine hydrochloride), poly(4-styrenesulfonic acid)) on microparticle size development [54]. 

The possibility of entrapment of drug molecules in an appropriate polymer matrix is mostly dependent on the miscibility of the substances. Generally, the bulky phase, i.e., polymer or solvent, may attract drug molecules by dispersive, polar, and hydrogen-bonding forces. In order to qualitatively and quantitatively describe the mentioned interactions, appropriate miscibility and solubility parameters, including the increments of dispersive polar and hydrogen bonding, have been introduced for numerous applications, especially the solubility of polymers in solvents and the dispersibility of bioactive compounds in solid or liquid polymeric matrices. The issues for CUR and ALG have been discussed according to calculated or determined solubility parameters in Electronic Supplementary Information (ESI) (see Table S1). Taking into account this discussion, CUR and ALG are compatible with each other, so curcumin may be molecularly dispersed (dissolved) in the polymer matrix; adsorption phenomena play a marginal role in this process. Moreover, CUR was used in the solid state for the encapsulation procedure to prevent uncontrolled leaching of the payload during the microparticle fabrication step. The specific microenvironment in the hydrogel core combined with the high thickness of the alginate matrix contributes to the physical entanglement of the CUR molecules between the cross-linked polysaccharide chains. Repulsive interactions between payload and alginate are considered minor, as CUR is a weak Bronsted acid, and therefore under slightly acidic conditions (pH ~ 6.8) it is in neutral form (pKa_H1_ ~ 8.5) [55].

Encapsulation efficiency (EE) is a crucial parameter that affects the quality of microparticles. This parameter describes the amount of the encapsulated drug divided by its initial amount introduced into the system during fabrication. Very low values of EE, e.g., less than 10–20%, may indicate that the drug molecules are chemically unstable in the system or are not entrapped in the carrier polymeric matrix and freely dissolved or dispersed in the solvent. The EE is also an important criterion for the successful delivery of therapeutics to sites of action because the value allows determining the suitability of a carrier with a given composition, i.e., optimal loading capacity and concentration of the delivered substance. On this basis, the kinetics of payload release from the carrier can be determined at each stage of the process to tailor the system to the therapeutic index. EE values for the developed microparticles are reported in Table 2 and show only slight differences between the values for uncoated microspheres and microcapsules coated with the modified polyelectrolytes **1**–**4**. For the CHIT containing microcapsules, the decrease of the EE in most cases was within the experimental error. A stronger decrease of the EE could be observed for PAH-containing capsules, in particular for CP2 and CP3. This may result from the higher porosity of the outer coating with the more hydrophobic polyelectrolyte. However, the EE of all fabricated microparticles was satisfactory for CUR encapsulation in microparticles.

Curcumin-loaded microcarriers coated with the grafted polyelectrolytes **1**–**4** (i.e., alkylene quaternary ammonium salt modified poly(acrylic acid)) were studied by FTIR and the most characteristic bands reflecting structural features were identified in the spectra, as shown in Figure S1. The spectra of other types of microparticles and curcumin are presented in the electronic supplementary material (Figure S2). FTIR analysis confirmed that CUR was successfully encapsulated in the alginate-based microparticles and CHIT or PAH, and antimicrobial decorated PAAs were adsorbed onto the core. The characteristic peaks of CUR were present in spectra of all types of microparticles. The signals at 3500 cm^−1^ correspond to the hydroxyl groups, and the peaks in the region between 1629–1635 cm^−1^ are associated with carbonyl (-C=O) groups of CUR [56]. The bands from 1575 to 1608 cm^−1^ are attributed to the C=C stretching vibrations of aromatic rings, and the bands located at 1130–1160 cm^−1^ are from ether (C-O-C) groups in the structure of CUR. The peaks at around 1500 cm^−1^ are attributed to stretching vibrations of C-C bands present in the aromatic ring, and signals observed in the region between 700–900 cm^−1^ are assigned to C-H bands of CUR [57]. Furthermore, FTIR spectra of microparticles revealed the presence of characteristic signals for sodium alginate. Wide bands in the range of 3070–3450 cm^−1^ are from hydroxyl (O-H) groups [58], the peaks at 1628 cm^−1^ are related to the asymmetric stretching vibration of carboxylate groups (-COOH), the signals at 1603 cm^−1^ correspond to the symmetric vibrations of carboxylate groups [58] and the signals around 1050–1110 cm^−1^ originate from C-O-C glycosidic bonds. Furthermore, FTIR analysis confirmed the presence of polyelectrolytes applied as microparticle coatings. The -NH_2_ group of CHIT and PAH showed a peak corresponding to the bending vibrations of N-H at 1540–1550 cm^−1^ [59]. The signals form C-N stretching vibrations, present in the structure of CHIT and the series of PAA-C(O)O-(CH_2_)_n_-N^+^(CH_3_)_3_(m) (n = 6, 12; m = 8, 10, 14%; products No. **1**–**4** in Table 1) were visible at 1460–1490 cm^−1^ [51], while the C-N stretching vibrations from the PAH structure were observed in the region from 1340 cm^−1^ to 1350 cm^−1^. The peaks in the range of 2842–2956 cm^−1^ resulted from the C-H stretching vibrations in alkyl chains of A-QAS-PEs. Furthermore, bands in 1660–1680 cm^−1^ related to the C=O bond in the amide groups confirm the formation of electrostatic complexes between the carboxylic group of ALG, PAA A-QAS-PEs, and the amino group of CHIT or PAH [52].

### 2.3. Features of the LbL Coatings Studied by QCM-D, Ellipsometry, and Zeta Potential Measurements

**Figure 5:**
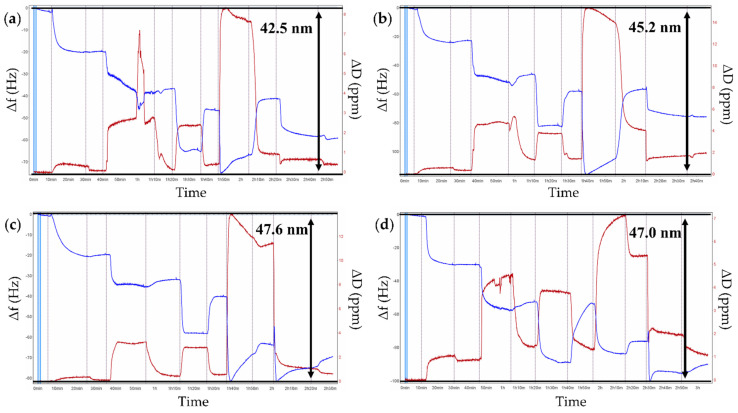
Exemplary graphs of adsorption kinetic measurements of polyelectrolyte multilayers monitored by QCM-D: (**a**) PEI/CUR/ALG/CHIT/1; (**b**) PEI/CUR/ALG/CHIT/2; (**c**) PEI/CUR/ALG/CHIT/3; (**d**) PEI/CUR/ALG/CHIT/4. Blue lines illustrate frequency shifts (Δf), red lines illustrate dissipation shifts (ΔD) of polyelectrolyte multilayers. Resonance frequency variations and dissipation variations are recorded as a function of time. Normalised frequency and dissipation shifts are shown for the seventh overtone.

Understanding the mechanism of polyelectrolyte deposition and the determination of film parameters, including thickness, mass, viscosity, and zeta potential, is fundamental for building multilayer films. The assembly of polyelectrolyte multilayer films on solid substrates was monitored in real time by QCM-D measurements to study adsorption kinetics and viscoelastic properties of the PE layers. Our experiments were performed in a particular manner to reproduce the sequence of the microparticles fabrication process. After PEI was used as an anchor layer at gold-coated QCM crystals, a crosslinked ALG layer loaded with CUR was deposited and crosslinked with CaCl_2_ to mimic the core of microparticles, then CHIT or PAH was adsorbed as the first coating layer and **1**–**4** functionalised polyelectrolytes (see Table 1) as the outer layer. Figure 5 and Figure S3 display the build-up of the multilayer film analysed in terms of normalised frequency changes (Δf) and energy dissipation shifts (ΔD) upon the adsorption of PEs. The typical decrease in the oscillation frequency observed during the deposition cycle indicates the increased mass on the sensor surface. The energy dissipation is related to the viscoelastic properties of the PE film: if the measured dissipation value is above 1 × 10^−6^ per 10 Hz frequency shift and spreads for overtones, the adsorbed layer is soft; if the dissipation value is less than 1 × 10^−6^ per 10 Hz frequency shift the deposited film is rigid [45]. 

When the surface of the quartz crystal was rinsed with distilled water, the frequency increased slightly due to the desorption of weakly bound polyelectrolyte molecules. Conversely, energy dissipation increased with adsorption of the subsequent PE layer and decreased during the rinsing step. Overall, the results showed in Figure 5 and Figure S3 confirm the successful assembly of each polyelectrolyte on gold-coated quartz sensors.

Analysis of the QCM-D results indicated that the Ca^2+^ crosslinked CUR-ALG layer formed rather stiff films as the energy dissipation was relatively low (4 × 10^−6^). Deposition of the polycation layer CHIT or PAH led to mass increase of the film and energy dissipation, presumably due to hydration. The subsequent adsorption of PAA or **1**–**4** polyelectrolytes caused a further decrease in frequency (adsorption) and stiffening of the film, which can be attributed to the collapse of the entire multilayer structure toward the interface during the adsorption of PAA and PAA-C(O)O-(CH_2_)_n_-N^+^(CH_3_)_3_(m) with the consequent formation of a rigid and thin structure. Notley et al. reported a similar result for the multilayered PAH/PAA build-up and observed a stiffening effect with the adsorption of PAA [60]. 

The QCM-D technique enabled us to quantitatively analyse the mass, thickness, and viscosity of adsorbed films applying the viscoelastic film model (see Section 3.9), as presented in Table 3. PAH forms a thicker and denser layer than CHIT, in agreement with the data reported in Table 2. In addition, for the CHIT system, the thickness of each bilayer system (CHIT/A-QAS-PEs) was lower than in the case of the bilayer PAH system (PAH/A-QAS-PEs). The thickness/mass of the outer layer was the lowest for the unmodified PAA and the highest for the functionalised polyelectrolytes with -(CH_2_)_12_- hydrocarbon chain irrespective of the polycation layer. These differences can be related to the higher molecular mass of those polyelectrolytes. The viscosity for both systems revealed the opposite trend to thickness or areal mass: the viscosity of the outer layers (PAA or PAA-C(O)O-(CH_2_)_n_-N^+^(CH_3_)_3_(m)) was higher than the viscosity of the polycation layers (CHIT or PAH). This means that the multilayer structure is more rigid with the PAA used as the final layer, as indicated by energy dissipation results. 

Spectroscopic ellipsometry allowed measuring the thickness of dry film layers, and the obtained results are listed in the Electronic Supplementary Material (Table S2). The ratio of the dry thickness of the CHIT layer (1.4 nm) and PAH layer (2.2 nm) agreed with the QCM-D results for wet thickness, as the CHIT layer was thinner than the PAH layer. The dry thickness of PAA-C(O)O-(CH_2_)_6_-N^+^(CH_3_)_3_-8% and PAA-C(O)O-(CH_2_)_6_-N^+^(CH_3_)_3_-10% was much higher than PAA-C(O)O-(CH_2_)_12_-N^+^(CH_3_)_3_-8% and PAA-C(O)O-(CH_2_)_12_-N^+^(CH_3_)_3_-14% regardless of the polycation layer underneath, unlike the thickness of the respective wet film layers. This may be the effect of weaker hydration in the dry state of modified polyelectrolytes containing more hydrophobic side chains. 

Since the formation of the multilayer coatings is mainly driven by the electrostatic interactions, we determined the zeta potentials of the polyelectrolyte building blocks of the films. The results are presented in Table 3. As expected, the values for cationic PEs exhibited a positive charge: ζ_CHIT_ = 40.9 mV, ζ_PAH_ = 35.7 mV. The zeta potential for unmodified PAA was ζ = −17.3 mV, whereas for **1**–**4** polyelectrolytes was, respectively, in the range of −35.8 to −26.7 mV, despite their functionalisation with positively charged quaternary amine groups. This difference may result from the lower pH of the PAA solution and the respective protonation of the carboxylic groups. The results obtained for the zeta potential for **1**–**4** polyelectrolytes confirm that the charge compensation did not occur, and the zeta potential value was negative. Our findings correspond to studies by Tartakovsky et al. on various poly(acrylic acid) amphoteric copolymers, i.e., comprising both anionic acrylate and cationic quaternary ammonium groups in one macromolecule in different ratios, which clearly showed that both ionic moieties are not complexed and are able to interact with charged surfaces [50]. It should be noted that the copolymer of acrylic acid (85%) and methylacrylamidopropyl trimethylammonium chloride (15%), synthesized and studied by the authors mentioned above, exhibited zeta potential of around −35 mV, related to our results for the poly(acrylic acid) derivative, that contains 14% cationic groups (value of zeta potential ca −35 mV, see Table 3). Similar absolute values of oppositely charged zeta potential enabled a successful sequential deposition of the PEs on both solid substrates and formation of the bilayer on hydrogel microparticles [61]. 

### 2.4. Curcumin Release Behavior 

**Figure 6:**
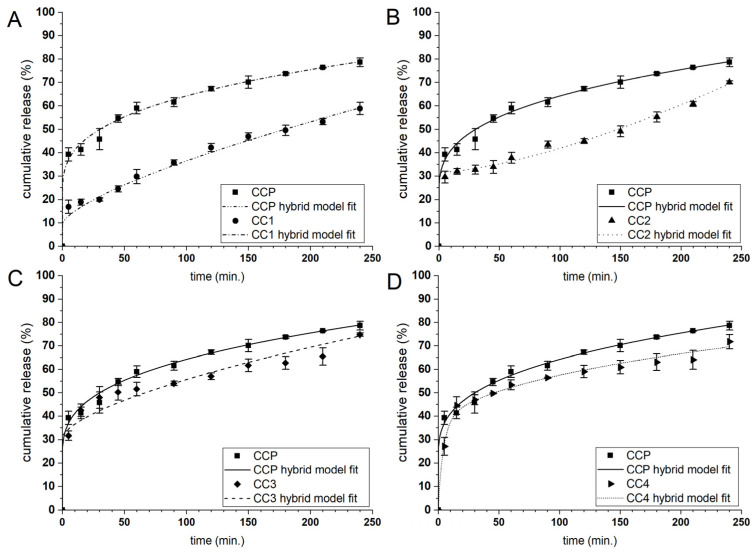
Curcumin release profiles in PBS solution (pH = 7.4) from CHIT coated microparticles with the antimicrobial decorated poly(acrylic acid): **1** (**A**); **2** (**B**); **3** (**C**) and **4** (**D**). The lines correspond to the fitting curves of the hybrid model to the payload release profiles.

**Figure 7:**
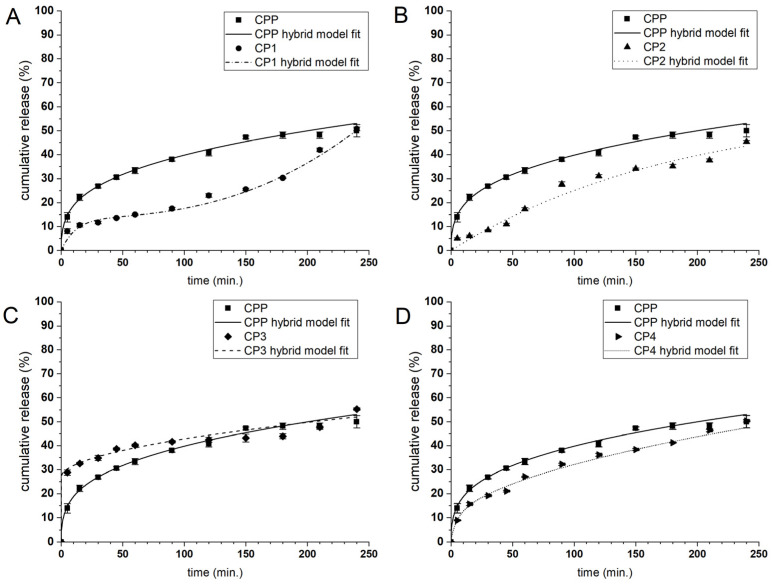
CUR release profiles in PBS solution (pH = 7.4) from microparticles of PAH system coated with antimicrobial decorated poly(acrylic acid): **1** (**A**); **2** (**B**); **3** (**C**) and **4** (**D**). The lines correspond to the fitting curves of the hybrid model to the payload release profiles.

The payload release mechanism under the required conditions plays a fundamental role in determining the ability of microcarriers to administer the active compound. The release profile of the enclosed substance depends on several factors, such as the payload and formulation characteristics and the fabrication process parameters. To determine the applicability of the designed microparticles as the curcumin carriers, their release behaviour was evaluated in a PBS solution at pH 7.4. The CUR release profiles of the microcarriers coated with the alkylene quaternary ammonium salt modified PAAs **1**–**4** (see Table 1) are presented in Figure 6 and Figure 7, while the CUR release profiles of the other types of microcarriers are presented in the Electronic Supplementary Material (Figure S4). CUR was released from the CHIT system microparticles faster than from those of the PAH system, behaviour that may be associated with the outer shell thickness because the CHIT system bilayers are thinner than those of the PAH system. The ALG matrix is sensitive to various factors such as pH and the ionic strength of the environment, so it was necessary to prepare uncoated and coated microparticles under neutral conditions so as not to cause uncontrolled leaching of CUR. In general, only a few systems exhibited significant initial burst release, possibly connected with some nonencapsulated curcumin present in the dispersion. A-QAS-PEs reduced burst releasing during the first 5 min of the release studies from ~50% to below 30% for CHIT-coated carriers and below 10% for PAH-coated carriers. Comparing the CUR release of all functionalised microcarriers, the highest release rate was observed for CC3 and CC4, which showed the initial burst release (42% and 45% of the payload, respectively), while such an effect was not observed for CC1 and CC2. In the case of the PAH system microparticles, the slowest release rate of CUR was noticed for CP1 and CP2. The CP3 and CP4 showed a higher release rate with a significant burst effect (22% and 15% of the payload, respectively). 

Based on these findings, we can conclude that **3** and **4** polyelectrolytes form thicker, more hydrophobic layers with higher compatibility with curcumin than the microcapsules interior; therefore, CUR can accumulate there during microcapsules formation and is swiftly released. On the other hand, polyelectrolytes **1** and **2** applied as the outer layer (on CHIT or PAH) form more compact and close-packed structures, which impede the payload release rate (cf. Figure 4). The CUR release profiles of CU and CC were similar, with initial burst release, but the faster CUR release kinetics were observed from the CC particles. The same trend was observed in a study describing the release of CUR from uncoated and CHIT-coated microparticles [27]. The CP microcarriers demonstrated a slower release rate with no burst effect.

We attempted to describe the CUR release kinetics from the microcapsules quantitatively by a phenomenologic model. We selected the hybrid model (H) that was previously found to be the most suitable for characterising the release of CUR. It combines first-order kinetics (FO) to describe the burst phase, and the Korsmeyer-Peppas (KP) model to describe the further stage of the release [54]. The fitting of the H model to the experimental data resulted in a good fit with a high R^2^ value (>0.953) (c.f., Figure 6, Figure 7 and Figure S3) for all compositions of the microparticles. In addition, the primary release mechanism after the burst phase could be determined for all microparticle types because the KP model can be used to study payload release from spherical, swellable hydrogel particles. The description of the release mechanism requires the examination of the release exponent coefficient (*n*): if *n* ≤ 0.43, the release depends on Fickian diffusion; if 0.43 < *n* < 0.85, the payload release is the result of diffusion and relaxation of the polymer chains; if *n* > 0.85 the release is controlled by the supercase II transport [62]. 

The results for the best-fit parameters of the H model for the CUR release profiles from all types of microparticles are shown in Table 4. The *n* value for uncoated and CHIT- or PAH-coated microparticles suggests diffusion was the primary CUR release mechanism. The single PAA layer as the microparticles coating did not modify the release mechanism. Similarly, for all chitosan-based bilayer systems, CUR diffusion out of the microparticles governs the release kinetics. On the other hand, the release of CUR from PAH/PE bilayer microparticles may also be associated with the relaxation of the polymer, as *n* > 0.43 was observed. These findings are in line with the QCM-D results showing that the **1**–**4** layers adsorbed on PAH form thicker layers (relatively to CHIT) that can relax during the release. In particular, for CP1 and CP2, the *n* values were higher than those for CP3 and CP4, suggesting that the side chains interaction of polyelectrolytes **1** and **2** with PAH “seals” the outer shell of the particles. Therefore, the CUR release requires the microparticles surface reorganisation (i.e., loosening of the polymer chains). Probably, the **3** and **4** grafted PAAs, deposited on PAH may form more porous shells with hydrophobic domains more favourable for the diffusion of CUR. Therefore, switching the payload release mechanism from diffusion to diffusion/relaxation of the polymer may be controlled by the thickness and stiffness of the PE bilayer films (Figure 5 and Table 3). 

### 2.5. Antimicrobial Activity of Bilayer CUR-Loaded Hydrogel Microcarriers with Antimicrobial Decorated Poly(acrylic acid) as the Outer Layer 

**Figure 8:**
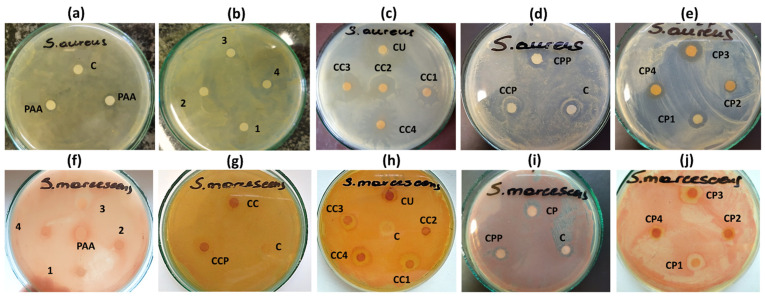
Photographs of sample inhibition zone test plates for PAA (**a**), decorated PEs 1, 2, 3, 4 (**b**), CU, CC1, CC2, CC3, CC4 microparticles (**c**), CCP, CPP microparticles (**d**), CP1, CP2, CP3, CP4 microparticles (**e**) against *S. aureus* and decorated PEs 1, 2, 3, 4 and PAA (**f**), CC, CCP microparticles (**g**), CU, CC1, CC2, CC3, CC4 microparticles (**h**), CP, CPP microparticles (**i**), CP1, CP2, CP3, CP4 microparticles (**j**) against *S. marcescens*. Distilled water or acetone was used as a control, C.

Multilayer microcarriers covered by A-QAS-PEs, which comprise cationic moieties in the outer layer structure, possess antimicrobial properties due to a general mechanism driven by electrostatic interactions between the cationic surface of the carrier and the negatively charged sites of the bacteria membrane. Such a mechanism can be influenced by the following parameters: (i) the composition of the antimicrobial microparticles; (ii) the nature of polyelectrolytes with antimicrobial function; (iii) the cell membrane composition; (iv) environmental parameters (i.e., temperature). In the present work, the antibacterial activity against *S. aureus* and *S. marcescens* of both antimicrobial decorated PAAs (**1**–**4**) and CUR-loaded hydrogel microparticles with **1**–**4** PAAs as the outer layer was determined using the disc diffusion method. The results expressed as an inhibition zone diameter (Φ) are given in Table 5 and illustrated in Figure 8. 

We observed a small inhibition zone for unmodified PAA, but the antimicrobial grafting of PAA increased inhibitory activity against both strains of bacteria. Although synthesised polyelectrolytes **1**–**4** used as the outer layer in the bilayer microparticles had negative zeta potentials, the presence of quaternary ammonium groups responsible for microbial cell damage significantly enhanced their antibacterial action. The negative zeta potential values of native A-QAS-PE (in globular conformation) indicated that they would interact electrostatically with the oppositely charged polyelectrolytes, namely CHIT and PAH. After deposition of A-QAS-PEs on the surface of the microparticles, the conformation of the polyelectrolytes changed to an extended coil, resulting in the surface quaternary ammonium groups being more available for interactions with the bacterial membrane. Under the conditions of the antibacterial tests (pH ~ 7.3), the quaternary ammonium groups were protonated (pKa ~ 9.8) [63]. Our assumptions were confirmed by the antimicrobial activity of the synthesized PEs, and the functionalized microparticles: A-QAS-PEs in solution were less effective than when they were used as an outer coating of particles. It is worth noting that a higher antimicrobial activity was observed against *S. aureus* (Φ = 10–12 mm) than against *S. marcescens* (Φ = 7 mm) for all studied bilayer hydrogel microcarriers with antimicrobial decorated PAA as the outer layer. It seems that Gram-positive bacteria may be more sensitive to the A-QAS-PEs representatives. These results are in good agreement with those reported by Belbekhouche et al., who also observed a higher inhibitory effect against Gram-positive bacteria than against Gram-negative bacteria [25]. This behaviour may be related to the structure of the bacterial cell envelope. Gram-positive bacteria have an inner membrane and a cell wall, whereas Gram-negative bacteria have an additional outer membrane in the cell envelope [1,62]. Moreover, the hydrophobicity and degree of substitution of cationic moieties influenced the antimicrobial activity of the new PEs. Furthermore, functionalized polyelectrolyte **1** and **2** showed a higher antibacterial efficacy against the strain of Gram-positive bacteria than **3** and **4**. This can be explained by the longer side alkyl chains in structures **3** and **4**, which form a hydrophobic barrier between positively charged units and the surface of the bacterial cell. Moreover, the shorter alkyl chains of **1** and **2** are more mobile in the microbial culture medium, and their cationic moieties can interact more effectively with the bacterial wall. The same tendency to increase the antimicrobial effect with decreasing length of the hydrophobic chain has been observed in other findings [15]. The higher degree of grafting improved the inhibition effect against Gram-positive bacteria, since the Φ was higher for decorated polyelectrolyte **4**. 

Our study indicated that all fabricated antimicrobial grafted bilayer microparticles exerted significant antibacterial activity against both strains. The results differed depending on the type of PE bilayer applied. We used acetone to inhibit the growth of bacteria as a negative control. The curcumin-loaded unmodified PAA coated microparticles (CCP and CPP) inhibited bacterial growth similar to acetone (Φ = 8 mm), while microparticles coated with **1**–**4** polyelectrolytes as the outer layer exerted a stronger effect on both tested bacteria strains (Φ = 11–20 mm). Therefore, the increase of the antibacterial effect cannot be associated with just the CUR release, but some synergistic effect of polyelectrolyte coating and curcumin is possible. A larger zone of growth inhibition was observed for *S. aureus* (Φ = 11–20 mm) than for *S. marcescens* (Φ = 11–12 mm), similar to the antibacterial activity of the PEs discussed above. Interestingly, in the case of CHIT system microcapsules, the diameter of the CC3 and CC4 inhibition zones against *S. aureus* were 2–3 mm wider than of the CC1 and CC2.

Similarly, the zone of inhibition against *S. marcescens* of CC3 and CC4 was 1 mm wider than of CC1 and CC2. This suggests that CC3 and CC4 showed greater antimicrobial efficacy than CC1 and CC2 in contrast to functionalised polyelectrolytes in aqueous solutions. These findings are in good agreement with the QCM-D results and the CUR release studies showing that the functionalised PEs **3** and **4** form a “loose” structure as the outer layer of the microcapsules and that the quaternary ammonium moieties are preferably exposed to effective interactions with the cell walls. On the other hand, positively charged units of decorated PEs **1** and **2** structures, which form more compact layers, are less accessible to bacterial cells. In the case of the PAH system microcarriers, the results slightly differed from those of the studied microparticles of the CHIT system. Their activity against *S. aureus* resulted in the inhibition zones: Φ = 13 mm for CP1 and Φ = 11 mm for CP2, while Φ = 15 mm for CP3 and Φ = 12 mm for CP4. The strain of *S. marcescens* strain turned out to be more resistant to these microparticles (Φ = 12 mm) than *S. aureus*. The results indicate that the functionalised CHIT system microparticles generated wider zones of inhibition for *S. aureus* than those of the PAH system, while for *S. marcescens,* similar antibacterial properties of both systems were observed. The stronger antibacterial effect of the CHIT system microparticles may result from the presence of CHIT in their structure, which has antibacterial activity on its own [64]. 

The above studies demonstrated that microcarriers decorated with the antibacterially functionalised PAA (A-QAS-PEs) show a stronger inhibitory effect on bacterial growth in comparison to polymers in aqueous solutions. This can be explained by the chemical structure of PEs and their hydrophobic chains, which partially hinder the exposure of quaternary ammonium moieties in the solution. Moreover, applying the A-QAS-PEs on the microparticle surface is likely to physically disrupt the polymer backbone and expose the terminal cationic moieties responsible for antimicrobial activity in the environment. 

## 3. Materials and Methods

### 3.1. Materials

Poly(acrylic acid) (molecular weight (Mw = 100 kDa) (PAA), trimethylamine (TMA), alginic acid sodium salt of medium viscosity (ALG), polyethylene imine (PEI) (Mw = 600–1000 kDa), poly(allylamine hydrochloride) (PAH) (Mw = 450 kDa), Span 80, Tween 80 and D_2_O (99.9% at D) were purchased from Sigma-Aldrich (Poznań, Poland). 12-bromododecan-1-ol (purity 99.13%) and 6-bromohexan-1-ol (purity 97.67%) were obtained from BLD Pharmatech Ltd. (Shanghai, China). N,N′-dicyclohexylcarbodiimide (DCC), 4-dimethylaminopyridine (DMAP) and chitosan (CHIT) (Mw = 100–300 kDa) were all synthetic grade and purchased from Acros Organics (Geel, Belgium). Curcumin (CUR) was obtained from Archem Sp. z o.o. (Kamieniec Wrocławski, Poland). Nutrient broth and Mueller-Hinton agar were purchased from Biocorp Polska Sp. z o.o. (Warszawa, Poland). Methanol and acetic acid were analytical grade and purchased from P.P.H.’ STANLAB’ Sp. J. (Lublin, Poland). Calcium chloride, dimethylsulfoxide (DMSO), hexane, and acetone (all chemicals of analytical grade) were obtained from Avantor Performance Materials (Gliwice, Poland).

### 3.2. Synthesis of the Poly(acrylic acid) Containing Alkylene Quaternary Ammonium Groupings

Under Steglich conditions, dodecyl and hexyl modified poly(acrylic acid) with ester linking groups were prepared as the raw material for the synthesis of side chain functionalised polyelectrolytes with antimicrobial moieties. Briefly: 13.89 mmol of carboxylic acid groups in poly(acrylic acid), 12-bromododecan-1-ol (4.17 mmol or 6.25 mmol for 8% or 14% substitution, respectively) or 6-bromohexan-1-ol (4.17 mmol or 6.25 mmol for 8% or 10% substitution, respectively), an appropriate amount of DCC (5.42 mmol or 8.13 mmol for 8% or 14% substitution for dodecyl functionalized poly(acrylic acid) or 5.42 mmol or 8.13 mmol for 8% or 10% substitution for hexyl functionalized poly(acrylic acid), respectively) and a catalytic amount of DMAP were dissolved in 50 mL of DMSO. The mixture was stirred at 22 °C for 48 h. After reaction completion, 1 mL of distilled water was added to the mixture to decompose unreacted DCC, followed by an additional 2 h of stirring. The precipitated by-product, dicyclohexylurea (DCU), was removed from the reaction mixture by filtration, followed by solution dialysis with distilled water (4 days, MWCO = 3500). The resulting solution was filtered, and the modified poly(acrylic acid) was isolated by lyophilisation. Next, the synthesised poly(acrylic acid) with an alkyl side chain was coupled with the following antimicrobial groups: dodecyl or hexyl modified poly(acrylic acid) (1.0 g) and sodium hydroxide (4.77 mmol or 2.97 mmol for substitution of 8% or 14% substitution for dodecyl functionalised poly(acrylic acid) or 5.73 mmol or 3.85 mmol for substitution of 8% or 10% substitution for hexyl functionalised poly(acrylic acid), respectively, were dissolved separately in methanol. The alcoholic polyelectrolyte solution was neutralised by dropping the methanolic sodium hydroxide solution into the prepared polymeric solution. The reaction mixture was left for 4 h at 22 °C, then 10 times the excess of TMA was added to the prepared solution and the whole mixture was left for 48 h at 4 °C. The organic solvent was removed under reduced pressure to obtain the desired product.

**PAA-C(O)O-(CH_2_)_6_-N^+^(CH_3_)_3_-8%**: Yield: 48%

FTIR/cm^−1^: 3359 (O-H, stretching), 2926 (C-H, stretching), 1702 (C=O, stretching-ester), 1664 (C=O, stretching-carboxylic acid), 1467 (C-N, stretching), 984 (C-O-C, stretching)

^1^H NMR/ppm, D_2_O: -CH(CO)CH_2_- (1.10–2.45, broad m), -CH_2_N- (3.60–3.65, m), -N(CH_3_)_3_ (3.31–3.33, s)

**PAA-C(O)O-(CH_2_)_6_-N^+^(CH_3_)_3_-10%**: Yield: 39%

FTIR/cm^−1^: 3365 (O-H, stretching), 2924 (C-H, stretching), 1703 (C=O, stretching-ester), 1666 (C=O, stretching-carboxylic acid), 1465 (C-N, stretching), 982 (C-O-C, stretching)

^1^H NMR/ppm, D_2_O: -CH(CO)CH_2_- (1.15–2.40, broad m), -CH_2_N- (3.60–3.65, m), -N(CH_3_)_3_ (3.32–3.35, s)

**PAA-C(O)O-(CH_2_)_12_-N^+^(CH_3_)_3_-8%**: Yield: 55%

FTIR/cm^−1^: 3327 (O-H, stretching), 2924 (C-H, stretching), 1708 (C=O, stretching-ester), 1654 (C=O, stretching-carboxylic acid), 1477 (C-N, stretching), 985 (C-O-C, stretching)

^1^H NMR/ppm, D_2_O: -CH(CO)CH_2_- (1.20–2.30, broad m), -CH_2_N- (3.56–3.61, m), -N(CH_3_)_3_ (3.25–3.30, s)

**PAA-C(O)O-(CH_2_)_12_-N^+^(CH_3_)_3_-14%**: Yield: 46%

FTIR/cm^−1^: 3325 (O-H, stretching), 2922 (C-H, stretching), 1706 (C=O, stretching-ester), 1643 (C=O, stretching-carboxylic acid), 1478 (C-N, stretching), 984 (C-O-C, stretching)

^1^H NMR/ppm, D_2_O: -CH(CO)CH_2_- (1.25–2.40, broad m), -CH_2_N- (3.52–3.59, m), -N(CH_3_)_3_ (3.24–3.31, s)

### 3.3. ^1^H NMR Spectroscopic Analysis

The new A-QAS-PEs were characterised by ^1^H NMR spectroscopy. The spectra were recorded using the Bruker AMX500 apparatus (Bruker, Billerica, MA, USA) at 25 °C. Before measurements, the A-QAS-PEs were dissolved in D_2_O (concentration 1–3%) and left overnight at room temperature. Chemical shifts are given in ppm (δ scale) and refer to HDO signal (4.71 ppm) with a spectral resolution of at least 0.730 Hz.

### 3.4. FTIR Spectroscopic Analysis

Fourier transform infrared (FTIR) spectra of A-QAS-PEs and fabricated microparticles were recorded with a spectrophotometer (IR Spirit, Shimadzu Corporation, Tokyo, Japan) equipped with a diamond crystal attenuated total reflection (ATR) accessory. Spectra were recorded in the range of 4000–400 cm^−1^, with a resolution of 2 cm^−1^ and 64 scans. 

### 3.5. Formation of Microspheres

An emulsion method was applied to prepare ALG-based microspheres encapsulating CUR, as previously described [27], with slight modifications. First, CUR was dispersed in a 1.5% ALG aqueous solution (1:100 *w*/*v*) containing 1% (*w*/*v*) Tween 80. The prepared solution was transferred to a round bottom reactor with hexane (1:4, *v*/*v*) containing 1% Span 80 (*w*/*v*) and emulsified at 900 rpm for 10 min to receive the first emulsion. Then, 0.6 M calcium chloride aqueous solution with 1% (*w*/*v*) Tween 80 was homogenised with hexane containing 1% Span 80 (*w*/*v*) (2:3, *v*/*v*) and the second emulsion was added dropwise to the first emulsion and stirred for 60 min. After complete crosslinking, ALG microbeads were withdrawn from the mixture, washed, and dried. 

### 3.6. Layer-by-Layer Microparticles Coating 

Microcapsules were formed by the LbL approach using CHIT or PAH as the polycation and PAA or PAA-C(O)O-(CH_2_)_n_-N^+^(CH_3_)_3_(m) as the polyanion. To obtain the first coating layer, the microspheres were dispersed in 0.4% CHIT solution (dissolved in 2% acetic acid) or 0.1% PAH aqueous solution by 3 min sonication (Polsonic) and stirred for 15 min. To deposit the second coating layer, the microparticles were dispersed by 3 min sonication in 0.1% PAA or PAA-C(O)O-(CH_2_)_n_-N^+^(CH_3_)_3_(m) aqueous solution (pH = 5) and then gently stirred for 10 min. After each polyelectrolyte deposition step, the microcapsules were filtered and washed with distilled water. The microparticles were finally collected, dried, and stored at ambient temperature. 

### 3.7. Morphology and Particle Size

The surface morphology of the fabricated microparticles was examined by scanning electron microscopy (SEM) (JSM-6601LV, JEOL, Tokyo, Japan) at an acceleration voltage of 4 to 13 kV. The size of the studied particles was determined using a polarisation microscope (Eclipse TE2000S, Nikon, Tokyo, Japan). The mean diameter of the microparticles was estimated as the mean diameter of at least 100 randomly selected particles. The polydispersity index (PdI) of the size distribution was characterised by the following equation:

(1) $$\mathrm{PdI}\;=\;{\left(\frac{{\bar{x}}_{S.D.}}{\bar{x}}\right)}^{2}$$

where: $\bar{x}$*_S.D._* is the standard deviation of the diameter of the microparticles and $\bar{x}$ is the mean number-average diameter of the microparticles [53]. 

The experiments were carried out in triplicate.

### 3.8. Encapsulation Efficiency 

To determine the encapsulation efficiency (EE) of the fabricated microparticles, they were first mechanically destroyed and then suspended in acetone for 48 h at room temperature with stirring. Then, the amount of CUR extracted from the particles was estimated using UV-Vis spectrophotometry (λ = 419 nm) (U-2900, Hitachi, Tokyo, Japan) and the appropriate calibration curve. The encapsulation efficiency was calculated using the following equation:

(2) $$\mathrm{EE}\;=\;\frac{m_{e}}{m_{i}}*\;100\%,$$

where *m_e_* is the mass of the CUR encapsulated in microparticles and *m_i_* is the initial mass of CUR used for encapsulation. Experiments were performed in triplicate. 

### 3.9. QCM-D Analysis

Multilayer build-up was analysed in situ with quartz crystal microbalance with dissipation monitoring (QSense Biolin Scientific, Gothenburg, Sweden). A 14 mm diameter piezoelectric quartz crystal covered on both sides with gold electrodes was used as a solid substrate for multilayer growth. Before use, the sensors were cleaned in an ultrasound bath using a 2% Hellmanex III solution (Hellma, Müllheim, Germany). The sensors were excited at their fundamental resonance frequency (4.95 MHz) and at the 3rd, 5th, 7th, 9th, and 11th overtones. The changes in frequency (Δf) and dissipation (ΔD) of all overtones as a function of time were monitored. The QCM-D experiments were performed at room temperature with a 0.150 mL/min flow rate. Measurements were started with distilled water to establish the baseline frequency of the sensor. Subsequently, the multilayer films were built by sequentially depositing the polyelectrolytes on the crystals. The initial layer of positively charged PEI (0.05%, *w*/*v*) was adsorbed as an anchor layer on the negatively charged gold sensors. After that, a curcumin-loaded alginate mixture (CUR-ALG) (0.15%, *w*/*v*) was deposited as a first layer and crosslinked with calcium ions to mimic the microparticle core. Then, CHIT (0.04%, *w*/*v*) or PAH and A-QAS-PE (0.05%, *w*/*v*) were adsorbed on the sensors to mimic microparticle coatings. After the deposition of each polyelectrolyte, the excess of unadsorbed molecules was rinsed with distilled water. The S1 Smartfit or B1 Broadfit models (QSense Dfind) were applied to estimate the soft layers’ parameters. They were established as the best-fit parameters for the frequency and dissipation values at the plateau reached at the rinsing step. For rigid films (ΔD < 1 × 10^−6^ per 10 Hz) [45], the mass of the adsorbed layer was calculated from the Sauerbrey equation shown below:

(3) $$\Delta m\;=\;-C\frac{\Delta f}{n}$$

where *C* is the crystal constant equal to 17.7 ng/cm^2^Hz and *n* is the overtone number.

### 3.10. Zeta Potential

Zeta potential measurements were conducted using LDE (Laser Doppler Electrophoresis). The experiments were performed using the Zetasizer Nano Series (Malvern Instruments, Malvern, UK). In calculations of zeta potential, the classical Helmholtz–Smoluchowski formula was used, taking the bulk values of dielectric constant, viscosity and neglecting effects of any surface conductivity and ion cloud relaxation. The aqueous solutions of CHIT (0.04%, *w*/*v*) or PAH, unmodified PAA and A-QAS-PE (0.05%, *w*/*v*) were used without pH adjustment. The measurement conditions for zeta potentials of CHIT, PAH, PAA and A-QAS-PEs corresponded to the conditions under which they are adsorbed at polyelectrolyte films (Q-CMD and ellipsometry) and at the surface of microparticles. Each value was obtained as an average of three subsequent runs of the instrument with at least three measurements. All experiments were performed at 22 °C.

### 3.11. Spectroscopic Ellipsometry Analysis

Spectroscopic ellipsometry was applied to measure the thickness of dry PE films. Before forming multilayer films, the 1 × 1 cm silicon wafers were washed with the piranha solution, rinsed with distilled water, boiled four times in distilled water, and stream-dried with air. After that, PEI (0.05%, *w*/*v*) was adsorbed onto the wafer as the precursor film was adsorbed onto the wafer. Next, CUR/ALG (0.15%, *w*/*v*) was deposited on a PEI film as the base layer, followed by crosslinking of ALG with calcium ions. Subsequently, CHIT (0.04%, *w*/*v*) or PAH (0.05%, *w*/*v*) was adsorbed on the ALG layer, and A-QAS-PE (0.05%, *w*/*v*) was deposited as the final layer. Each PE adsorption lasted 20 min, followed by washing with distilled water to remove excess of unadsorbed PE. Finally, the samples were dried with air. The thickness and optical parameters of the films were estimated by fitting the Cauchy optical model to the measured wavelength dependence of the ellipsometric angles Δ and Ψ as described previously [61]. 

### 3.12. Release Profiles

The kinetics of CUR release from the fabricated microparticles were studied in phosphate buffer saline (PBS) (pH = 7.4) at 37 °C for 24 h. Initially, microcarriers were soaked in PBS (1:40, *w*/*v*) and transferred into a cellulose bag. The bag was then closed, immersed in 8 mL of PBS medium, and stirred (1000 rpm) to initiate the release of CUR. At predetermined time intervals, the release medium was withdrawn and replaced with the same volume of fresh medium. Finally, the amount of CUR was estimated by spectrophotometry (λ = 419 nm) (U-2900, Hitachi, Tokyo, Japan) and an appropriate calibration curve. The results obtained were expressed as the percentage of cumulative CUR release over time. Experiments were performed in triplicate. The CUR release profiles of microparticles were described using the following hybrid model [54]:

(4) $$\frac{M_{t}}{M_{\infty }}\;=\;\frac{M_{1}}{M_{\infty }}\;\left(1-e^{\left(-k_{1}t\right)}\right)\;+\;k_{2}t^{n_{1}}$$

where: *M* is the amount of CUR released during the burst phase; *k*_1_ is a first-order rate constant and *k*_2_ is a Korsmeyer-Peppas release constant; *n* is the release exponent. The model was fitted to the experimental data using OriginPro 9.0 software (OriginLab Corporation, Northampton, MA, USA). 

### 3.13. Antimicrobial Activity—Disc Diffusion Method

The antimicrobial activities of the A-QAS-PEs and the microparticles coated with A-QAS-PEs were tested against the Gram-positive bacterial strain (Staphylococcus aureus PCM 2054) and the Gram-negative strain (Serratia marcescens PCM 549) using the disc diffusion method. The bacterial strains were cultivated in nutrient broth for 24h and each bacterial suspension used as inoculum was adjusted to 0.5 McFarland standard. The Petri dishes were dried for 10 min before being used in the antimicrobial activity test. Sterile filter paper discs (6 mm diameter) were impregnated with a 10 µL solution of polyelectrolytes or microparticles suspension (C = 10 mg/mL). The discs were placed on Mueller-Hinton agar plates inoculated with the bacteria. Sterile double-distilled water or acetone was used as a negative control for polyelectrolytes or microparticles, respectively. All plates were left for 15 min at room temperature to allow diffusion. The plates were then incubated at 37 °C for 24 h. After incubation, the diameters of the inhibition zones formed around the discs were measured. Experiments were performed in triplicate. 

## 4. Conclusions

We successfully engineered and characterised four types of new antimicrobial grafted polyelectrolytes, i.e., alkylene quaternary ammonium salt functionalised poly(acrylic acid) (PAA) derivatives, which were found to be suitable to coat as the outer layer a series of curcumin (CUR)-loaded hydrogel microparticles via the layer-by-layer (LbL) technique. The fabricated bilayered microcarriers consisted of sodium alginate (ALG) as the core component, biocompatible/biodegradable chitosan (CHIT) or synthetic poly(allylamine hydrochloride) (PAH) provided the first coating layer and a newly synthesised antimicrobial decorated PAA, deposited as the outer layer, provided the functional coating. Differences in their chemical structure influenced their physicochemical and biological properties. In particular, the antimicrobial grafted PAAs could form films with different viscoelastic properties that directly resulted from their hydrophobicity. All fabricated bilayer CUR-loaded microcarriers, coated by alkylene quaternary ammonium salt grafted PAAs, showed stronger antibacterial activity against *S. aureus* and *S. marcescens* than the antimicrobial decorated PAAs on their own. We also observed that the release of CUR from the functionalised microcapsules could be controlled by the type of the new PE used as an outer layer. 

Therefore, modification of the microcarrier surface with PEs containing cationic moieties made it possible to fabricate new microsystems with desirable antimicrobial functions and, additionally, they provided desirable possibilities of the payload controlled release. The effective antimicrobial activity and drug release features of the designed microsystems open a new path for developing microcarriers with improved therapeutic applications, e.g., in the treatment of bacterial infectious diseases. Our findings could provide guidance for future, new, improved alkylene quaternary ammonium salt grafted polyelectrolytes (A-QAS-PEs)—products comprising even higher degrees of substitution for quaternary ammonium groups—with many tailored applications in numerous fields.

## Figures and Tables

**Table 1:** Structure and abbreviations of the alkylene quaternary ammonium salt decorated PAA derivatives.

No	Structure	(CH_2_)_n_	% Degree of Substitution (m)	Abbreviation
**1**	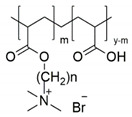	(CH_2_)_6_	8	PAA-C(O)O-(CH_2_)_6_-N^+^(CH_3_)_3_-8%
**2**			10	PAA-C(O)O-(CH_2_)_6_-N^+^(CH_3_)_3_-10%
**3**		(CH_2_)_12_	8	PAA-C(O)O-(CH_2_)_12_-N^+^(CH_3_)_3_-8%
**4**			14	PAA-C(O)O-(CH_2_)_12_-N^+^(CH_3_)_3_-14%

**Table 2:** Abbreviations, composition, and characteristics of the formulated CUR-loaded microparticles.

CHIT System					PAH System				
Micro-Particles	Composition of PE Coatings	MD ^a^ [µm]	PdI ^b^	EE ^c^ [%]	Micro-Particles	Composition of PE Coatings	MD ^a^ [µm]	PdI ^b^	EE ^c^ [%]
CU	uncoated	45.9 ± 4.9	0.225	56.1 ± 3.8	CU	uncoated	45.9 ± 4.9	0.225	56.1 ± 3.8
CC	CHIT	48.9 ± 5.1	0.159	58.5 ± 4.8	CP	PAH	60.7 ± 6.1	0.108	52.2 ± 1.8
CCP	CHIT/PAA	47.3 ± 5.9	0.208	56.7 ± 6.4	CPP	PAH/PAA	59.0 ± 5.4	0.072	60.5 ± 2.5
CC1	CHIT/1 *	46.2 ± 4.8	0.101	48.7 ± 2.3	CP1	PAH/1 *	57.2 ± 4.0	0.044	53.8 ± 3.2
CC2	CHIT/2 *	50.9 ± 6.0	0.121	53.9 ± 2.9	CP2	PAH/2 *	56.9 ± 6.3	0.085	45.8 ± 1.9
CC3	CHIT/3 *	50.0 ± 4.4	0.122	48.3 ± 3.0	CP3	PAH/3 *	59.5 ± 5.7	0.066	44.6 ± 1.1
CC4	CHIT/4 *	48.1 ± 5.1	0.103	55.4 ± 1.7	CP4	PAH/4 *	58.9 ± 6.2	0.078	56.2 ± 2.7

^a^ MD-mean diameter; ^b^ PdI-polydispersity index; ^c^ EE-encapsulation efficiency; * **1**–**4**: alkylene quaternary ammonium salt functionalised poly(acrylic acid) (for details, see Table 1). The results are expressed as mean ± S.D.

**Table 3:** Characteristics of the PE layers analysed with the QCM-D measurements.

	PE Layer	Thickness [nm]	Areal Mass [µg/cm^2^]	Viscosity [g/ms^2^]	Zeta Potential [mV]
CHIT system	CHIT	17.9 ± 2.2	1.9 ± 0.2	2.5 ± 0.4	41 ± 5.1
	PAA	9.6 ± 3.0	1.0 ± 0.3	6.1 ± 0.9	−17.3 ± 1.0
	PAA-C(O)O-(CH_2_)_6_-N^+^(CH_3_)_3_-8%	10.6 ± 0.1	1.1 ± 0.1	7.9 ± 1.8	−31 ± 5
	PAA-C(O)O-(CH_2_)_6_-N^+^(CH_3_)_3_-10%	13.3 ± 0.5	1.3 ± 0.1	4.9 ± 2.1	−27 ± 1.1
	PAA-C(O)O-(CH_2_)_12_-N^+^(CH_3_)_3_-8%	15.7 ± 3.9	1.7 ± 0.4	7.3 ± 1.0	−32.5 ± 2.0
	PAA-C(O)O-(CH_2_)_12_-N^+^(CH_3_)_3_-14%	15.1 ± 1.7	1.6 ± 0.2	7.1 ± 2.7	−36 ± 2.5
PAH system	PAH	22.8 ± 3.4	2.8 ± 0.6	2.1 ± 0.5	36 ± 2.8
	PAA	13.2 ± 2.5	1.3 ± 0.7	3.4 ± 0.4	−17.3 ± 1.0
	PAA-C(O)O-(CH_2_)_6_-N^+^(CH_3_)_3_-8%	14.7 ± 1.3	1.5 ± 0.2	4.9 ± 0.6	−31 ± 5
	PAA-C(O)O-(CH_2_)_6_-N^+^(CH_3_)_3_-10%	16.7 ± 1.7	1.8 ± 0.3	5.4 ± 1.6	−26.7 ± 1.1
	PAA-C(O)O-(CH_2_)_12_-N^+^(CH_3_)_3_-8%	20.5 ± 3.5	2.1 ± 0.4	3.2 ± 0.6	−33 ± 1.7
	PAA-C(O)O-(CH_2_)_12_-N^+^(CH_3_)_3_-14%	18.9 ± 4.0	1.9 ± 0.6	3.1 ± 0.9	−36 ± 2.4

**Table 4:** Kinetic parameters of the hybrid model obtained from the fit to the experimental release profiles of CUR from the microparticles of CHIT and PAH systems.

Microparticles		M	k_1_	k_2_	n	R^2^
CHIT system	CU	0.005	1.00 × 10^−3^	3.2 × 10^−1^	0.18	0.977
	CC	0.005	1.00 × 10^−3^	3.9 × 10^−1^	0.16	0.980
	CCP	0.005	1.00 × 10^−3^	2.3 × 10^−1^	0.22	0.992
	CC1	1.420	1.00 × 10^−3^	9.3 × 10^−1^	0.20	0.995
	CC2	1.127	1.00 × 10^−3^	1.8 × 10^−1^	0.15	0.973
	CC3	0.005	1.00 × 10^−3^	2.3 × 10^−1^	0.20	0.985
	CC4	0.005	1.00 × 10^−3^	2.2 × 10^−1^	0.21	0.985
PAH system	CP	1.000	1.94 × 10^−3^	1.4 × 10^−1^	0.18	0.985
	CPP	0.005	1.00 × 10^−3^	9.2 × 10^−2^	0.31	0.997
	CP1	0.005	1.00 × 10^−3^	5.8 × 10^−3^	0.79	0.953
	CP2	0.005	1.00 × 10^−3^	1.1 × 10^−2^	0.67	0.987
	CP3	0.005	1.00 × 10^−3^	1.9 × 10^−1^	0.47	0.969
	CP4	0.005	1.00 × 10^−3^	4.2 × 10^−2^	0.45	0.997

M—the amount of CUR released during the burst phase; k_1_—first-order rate constant, k_2_—Korsmeyer-Peppas release constant; n—the release exponent; R^2^—coefficient of determination.

**Table 5:** Diameters of inhibition zone (mm) for A-QAS-PEs and microcarriers coated with A-QAS-PEs against *S. aureus* and *S. marcescens.*

	Studied Sample	Zone of Inhibition (mm)	
		*S. aureus*	*S. marcescens*
Polyelectrolyte	control	-	-
	PAA	9 ± 0.8	-
	PAA-C(O)O-(CH_2_)_6_-N^+^(CH_3_)_3_-8%	12 ± 0.6	7 ± 0.1
	PAA-C(O)O-(CH_2_)_6_-N^+^(CH_3_)_3_-10%	12 ± 0.6	7 ± 0.1
	PAA-C(O)O-(CH_2_)_12_-N^+^(CH_3_)_3_-8%	10 ± 0.6	7 ± 0.1
	PAA-C(O)O-(CH_2_)_12_-N^+^(CH_3_)_3_-14%	11 ± 0.6	7 ± 0.1
Microparticle	control	8 ± 0.2	8 ± 0.3
	CCP	8 ± 0.3	8 ± 0.2
	CC1	16 ± 1.0	12 ± 0.8
	CC2	17 ± 0.5	12 ± 0.7
	CC3	18 ± 0.6	11 ± 0.6
	CC4	20 ± 0.6	12 ± 0.8
	CPP	8 ± 0.4	8 ± 0.4
	CP1	13 ± 0.8	12 ± 0.7
	CP2	11 ± 0.6	12 ± 0.8
	CP3	15 ± 1.0	12 ± 0.6
	CP4	12 ± 0.6	12 ± 0.7

The results are expressed as mean ± S.D.

## Data Availability

The data presented in this study are available within the article.

## Supplementary Materials

The following are available online. Figure S1: FTIR spectra of CUR-loaded microparticles decorated with A-QAS-PEs: CHIT system (a) and PAH system (b). Figure S2: FTIR spectra of CUR, CU, CC, and CP. Figure S3: Adsorption kinetic measurements of polyelectrolyte multilayers monitored by QCM-D: (a) PEI/CUR/ALG/CHIT/1; (b) PEI/CUR/ALG/CHIT/2; (c) PEI/CUR/ALG/CHIT/3; (d) PEI/CUR/ALG/CHIT/4; (e) PEI/CUR/ALG/CHIT/PAA; (f) PEI/CUR/ALG/PAH/PAA; (g) PEI/CUR/ALG/PAH/1; (h) PEI/CUR/ALG/PAH/2; (i) PEI/CUR/ALG/PAH/3; (j) PEI/CUR/ALG/PAH/4. Blue lines illustrate frequency shifts (Δf), while red lines illustrate dissipation shifts (ΔD) of polyelectrolyte multilayers. Resonance frequency variations and dissipation variations are recorded as functions of time. Figure S4: CUR release profiles from CU (squares), CC (circles), and CP (triangles) microparticles. The lines correspond to the fitting curves of the hybrid model to the payload release profiles. Table S1: Values of dispersion forces (δ_d_), polar forces (δ_p_) and hydrogen bonding (δ_h_) for the solubility parameters of curcumin and alginate as well as Euclidean distance (solubility parameter difference, ∆δ). Table S2: Film thickness characterisation by spectroscopic ellipsometry.

## Sample Availability

Samples of the polyelectrolytes are available from the authors.
