# Supplementary material for: Multilayered Curcumin-Loaded Hydrogel Microcarriers with Antimicrobial Function
Source: Molecules. 2022 Feb 19;27(4):1415. doi: 10.3390/molecules27041415 (PMC8875356; doi:10.3390/molecules27041415)
Supplement: Supplementary file 1 [file molecules-27-01415-s001.zip › molecules-1592828-supplementary.pdf]

Electronic Supplementary Material (ESI)

Multilayered curcumin-loaded hydrogel microcarriers with antimicrobial function

Weronika Szczęsna[1], Marta Tsirigotis-Maniecka[1], Łukasz Lamch[1], Lilianna Szyk-Warszyńska[2], Ewa Zboińska[3], Piotr Warszyński[2,*], Kazimiera A. Wilk[1,*]

[1] Department of Engineering and Technology of Chemical Processes, Faculty of Chemistry, Wrocław University of Science and Technology, 50-370 Wrocław, Poland; weronika.szczesna@pwr.edu.pl; marta.tsirigotis@pwr.edu.pl; lukasz.lamch@pwr.edu.pl; kazimiera.wilk@pwr.edu.pl

[2] Jerzy Haber Institute of Catalysis and Surface Chemistry, Polish Academy of Sciences, 30-239 Kraków, Poland; ncszyk@cyf-kr.edu.pl; ncwarszy@cyf-kr.edu.pl

[3] Department of Organic and Medicinal Chemistry, Faculty of Chemistry, Wrocław University of Science and Technology, 50-370 Wrocław, Poland; ewa.zboinska@pwr.edu.pl

* Correspondence: ncwarszy@cyf-kr.edu.pl; kazimiera.wilk@pwr.edu.pl

Table of Contents

Figure S1. FTIR spectra of CUR-loaded microparticles decorated with A-QAS-PEs: CHIT system (**a**) and PAH system (**b**).

Figure S2. FTIR spectra of CUR, CU, CC, and CP.

Figure S3. Adsorption kinetic measurements of polyelectrolyte multilayers monitored by QCM-D: (**a**) PEI/CUR/ALG/CHIT/**1**; (**b**) PEI/CUR/ALG/CHIT/**2**; (**c**) PEI/CUR/ALG/CHIT/**3**; (**d**) PEI/CUR/ALG/CHIT/**4**; (**e**) PEI/CUR/ALG/CHIT/PAA; (**f**) PEI/CUR/ALG/PAH/PAA; (**g**) PEI/CUR/ALG/PAH/**1**; (**h**) PEI/CUR/ALG/PAH/**2**; (**i**) PEI/CUR/ALG/PAH/**3**; (**j**) PEI/CUR/ALG/PAH/**4**. Blue lines illustrate frequency shifts (Δf), while red lines illustrate dissipation shifts (ΔD) of polyelectrolyte multilayers. Resonance frequency variations and dissipation variations are recorded as functions of time.

Figure S4. CUR release profiles from CU (squares), CC (circles), and CP (triangles) microparticles. The lines correspond to the fitting curves of the hybrid model to the payload release profiles.

Table S1. Values of dispersion forces (δ_d), polar forces (δ_p) and hydrogen bonding (δ_h) for the solubility parameters of curcumin and alginate as well as Euclidean distance (solubility parameter difference, $\Delta\delta$).

Table S2. Film thickness characterization by spectroscopic ellipsometry.

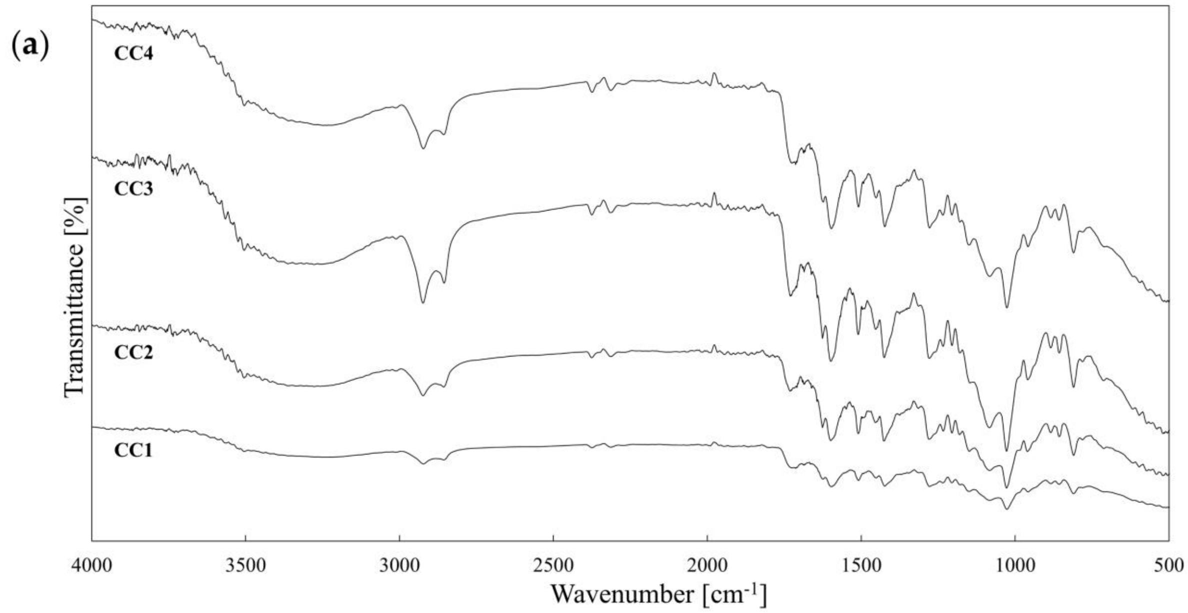

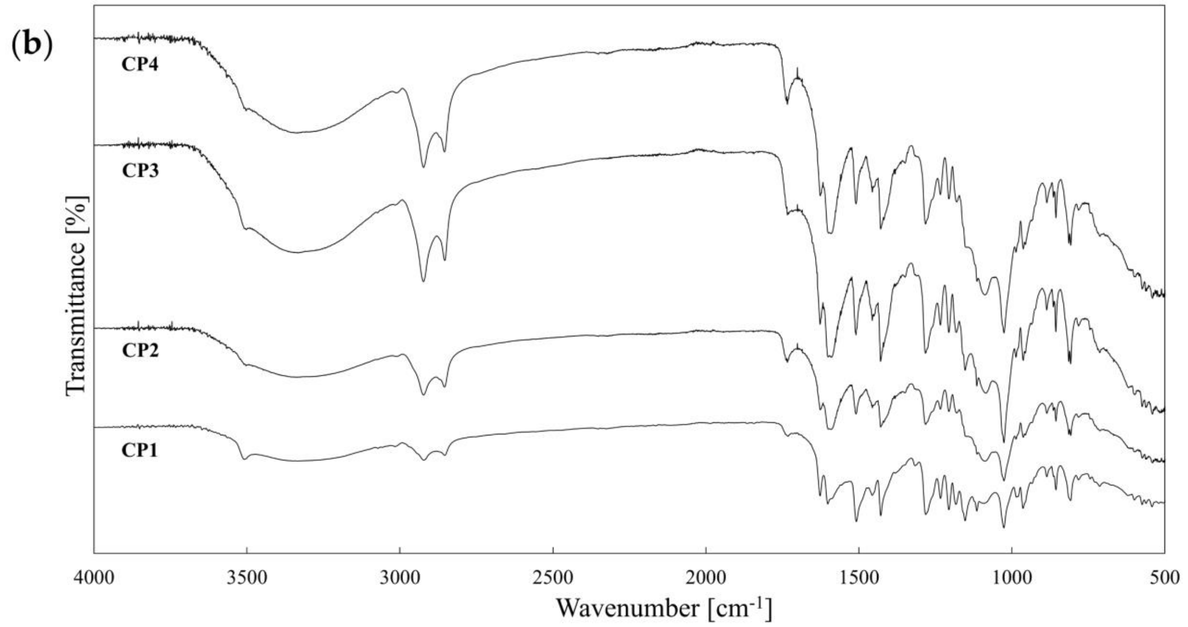

Figure S1. FTIR spectra of CUR-loaded microparticles decorated with A-QAS-PAAs: CHIT system (**a**) and PAH system (**b**).

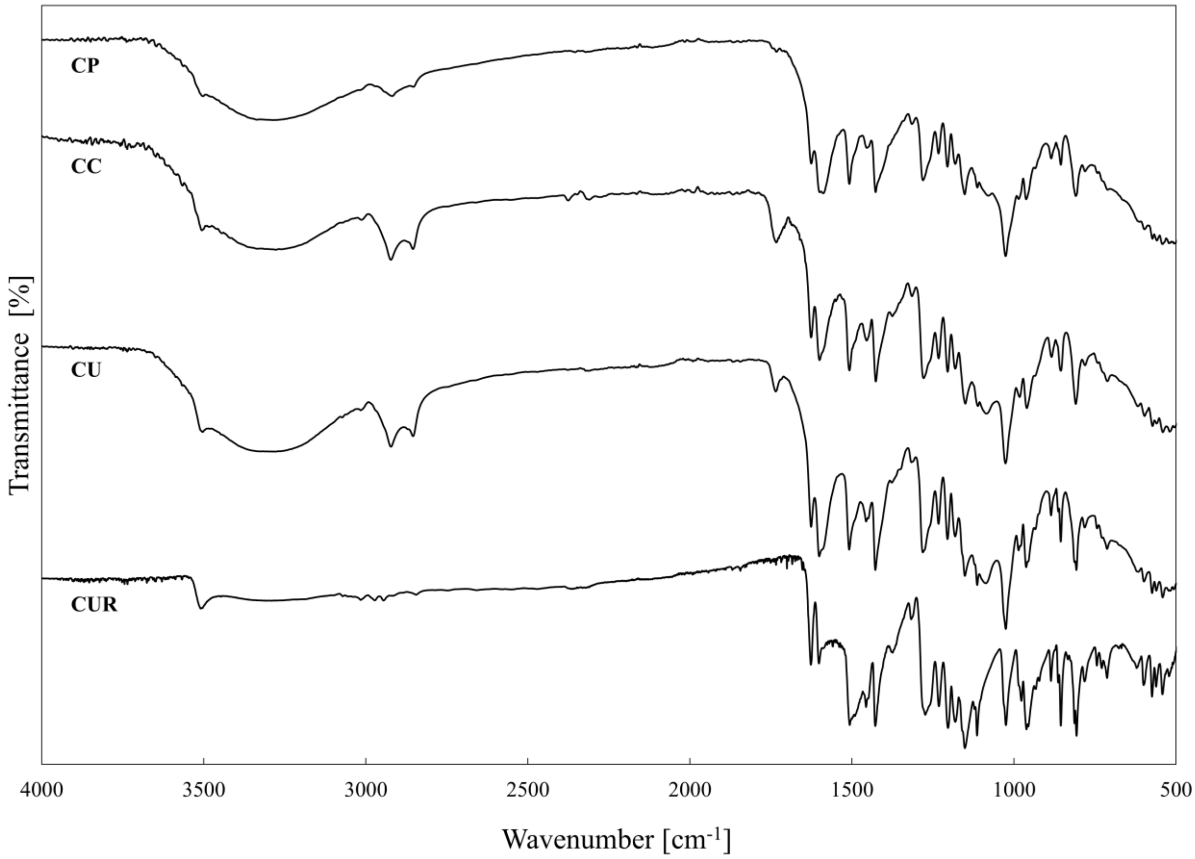

Figure S2. FTIR spectra of CUR, CU, CC and CP.

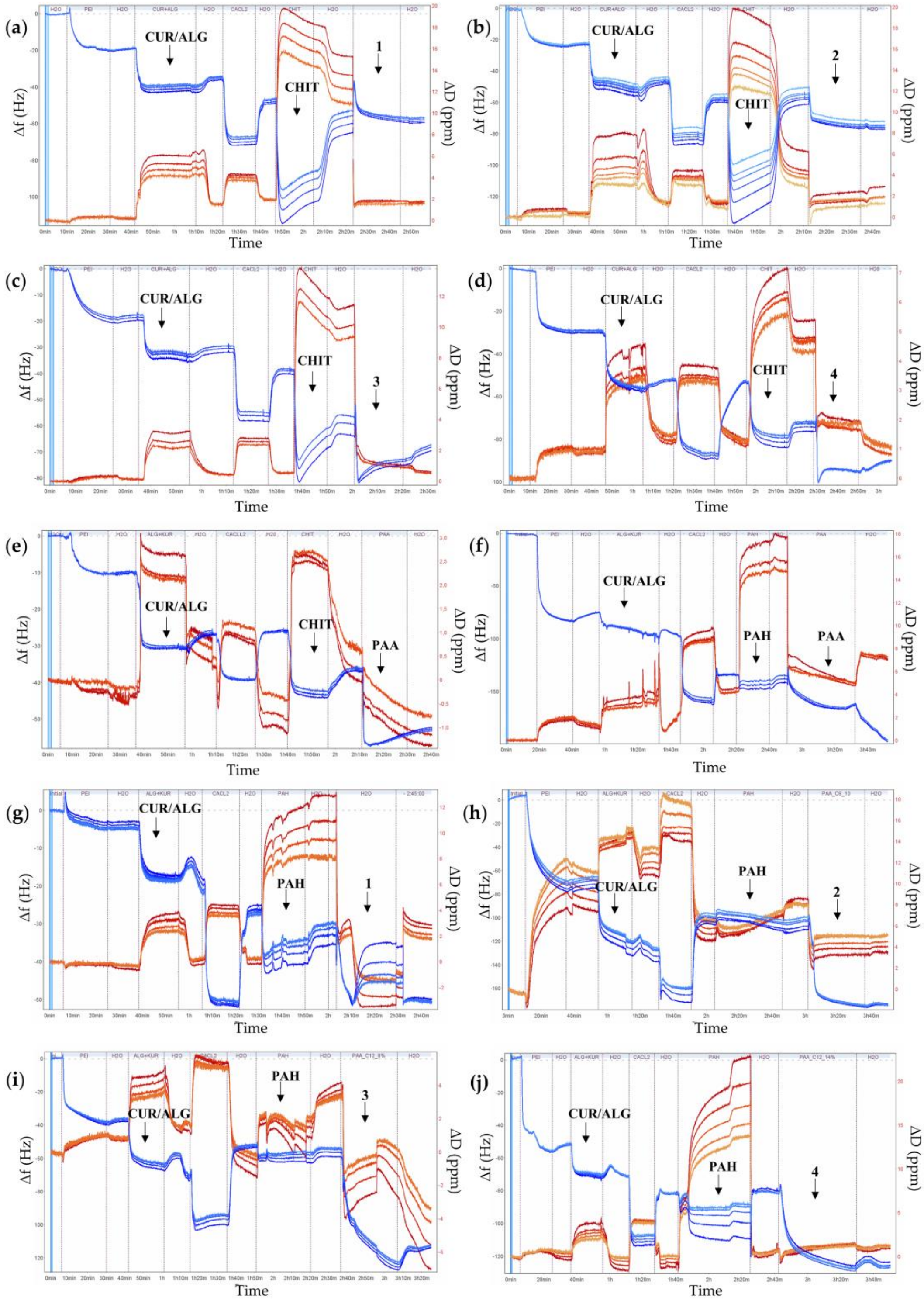

Figure S3. Adsorption kinetic measurements of polyelectrolyte multilayers monitored by QCM-D: (**a**) PEI/CUR/ALG/CHIT/**1**; (**b**) PEI/CUR/ALG/CHIT/**2**; (**c**) PEI/CUR/ALG/CHIT/**3**; (**d**) PEI/CUR/ALG/CHIT/**4**; (**e**) PEI/CUR/ALG/CHIT/PAA; (**f**) PEI/CUR/ALG/PAH/PAA; (**g**) PEI/CUR/ALG/PAH/**1**; (**h**) PEI/CUR/ALG/PAH/**2**; (**i**) PEI/CUR/ALG/PAH/**3**; (**j**) PEI/CUR/ALG/PAH/**4**. Blue lines illustrate frequency shifts (Δf), while red lines illustrate dissipation shifts (ΔD) of polyelectrolyte multilayers. Resonance frequency variations and dissipation variations are recorded as functions of time.

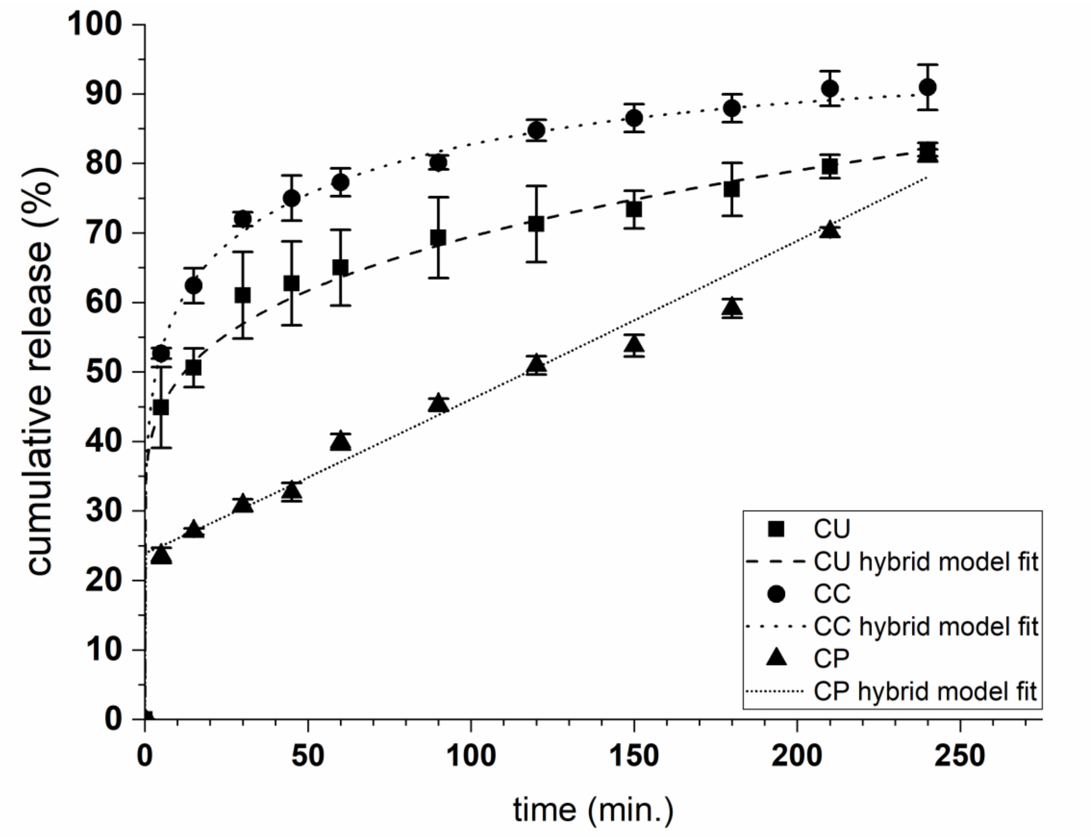

Figure S4. Release profiles of CUR from CU (squares), CC (circles), and CP (triangles) microparticles. Lines correspond to the fitting curves of the hybrid model to the payload release profiles.

Solubility studies of curcumin in alginate

In order to study the compatibility between curcumin and alginate – the active payload and the polymer matrix in our system – calculations of solubility and miscibility parameters were performed. This approach has been widely used for different drug-polymer pairs and is considered a very efficient tool for predicting and explaining various phenomena in such systems [1- 4]. The components of the dispersion forces (δ_d), polar forces (δ_p) and the hydrogen bonding (δ_h) for the solubility parameters of curcumin ($\delta^{(C)}$) and alginate ($\delta^{(A)}$) were calculated according to the group contribution method of Hoftyzer-van Krevelen [1]:

$$\delta_d = \frac{\sum F_{di}}{V} \tag{1}$$

$$\delta_p = \frac{\sqrt{\sum F_{pi}^2}}{V} \tag{2}$$

$$\delta_h = \sqrt{\frac{\sum F_{hi}}{V}} \tag{3}$$

where: F_{di}, F_{pi} and F_{hi} denote increments for dispersive, polar, and hydrogen bonding forces, respectively, while V denotes molar volumes. The Euclidean distance (solubility parameter difference, Δδ) was calculated according to the following equation [4]:

$$\Delta\delta = \sqrt{(\delta_d^{(C)} - \delta_d^{(A)})^2 + 0.25(\delta_p^{(C)} - \delta_p^{(A)})^2 + 0.25(\delta_h^{(C)} - \delta_h^{(A)})^2} \quad (4)$$

The calculated values of the appropriate components for dispersion forces, polar forces and hydrogen bonding for the solubility parameters of curcumin and alginate, as well as the value of Euclidean distance, were collected in Table S1.

Table S1. Values of dispersion forces (δ_d), polar forces (δ_p) and hydrogen bonding (δ_h) for the solubility parameters of curcumin and alginate as well as Euclidean distance (solubility parameter difference, Δδ).

CUR			ALG			Euclidean distance
$\delta_d^{(C)}$	$\delta_p^{(C)}$	$\delta_h^{(C)}$	$\delta_d^{(A)}$	$\delta_p^{(A)}$	$\delta_h^{(A)}$	Δδ
$MPa^{0.5}$	$MPa^{0.5}$	$MPa^{0.5}$	$MPa^{0.5}$	$MPa^{0.5}$	$MPa^{0.5}$	$MPa^{0.5}$
22.3	5.6	14.1	24.8	14.2	28.3	8.67

The Euclidean distance – or the difference in the solubility parameter difference, Δδ – may play a role as an indicator of compatibility between two different substances. A low value of Δδ indicate that chemicals are miscible with each other, i.e., soluble (in the case of solvents and substances dissolved in them) or dispersible (for solid mixtures). Generally, dispersions in polymers with $\Delta\delta < 10$ $MPa^{0.5}$ are considered to form a mixture with a high degree of compatibility, while $\Delta\delta \approx 5$ $MPa^{0.5}$ (or 5.6 $MPa^{0.5}$) are regarded as a border between solubility and insolubility in simple organic solvents. Our system, curcumin in alginate, is characterized by a Euclidean distance of around 8.67 $MPa^{0.5}$, so this pair may be considered miscible. This fact is in good agreement with the results of our studies – it has been found that the alginate core comprises appropriate host material for curcumin entrapment. Furthermore, the dispersion forces (δ_d), polar forces (δ_p) and hydrogen bonding (δ_h) for the solubility parameters, calculated according to the Hoftyzer-van Krevelen method, are sufficiently accurate for further calculations: the experimental values of $\delta_d^{(C)} = 18.8$ $MPa^{0.5}$, $\delta_p^{(C)} = 7.7$ $MPa^{0.5}$ and $\delta_p^{(C)} = 11.1$ $MPa^{0.5}$. [2]

Table S2. Film thickness characterization by spectroscopic ellipsometry.

	**PE layer**	**thickness [nm]**		**PE layer**	**thickness [nm]**
	CUR/ALG	1.6±0.1		CUR/ALG	1.6±0.1
CHIT system	CHIT	1.4±0.1	PAH system	PAH	2.2±0.2
	PAA	0.8±0.2		PAA	1.3±0.2
	PAA-C(O)O-$(CH_2)_6$-$N^+(CH_3)_3$-8%	4.7±0.2		PAA-C(O)O-$(CH_2)_6$-$N^+(CH_3)_3$-8%	7.2±0.7
	PAA-C(O)O-$(CH_2)_6$-$N^+(CH_3)_3$-10%	6.4±1.4		PAA-C(O)O-$(CH_2)_6$-$N^+(CH_3)_3$-10%	7.6±0.3
	PAA-C(O)O-$(CH_2)_{12}$-$N^+(CH_3)_3$-8%	1.1±0.2		PAA-C(O)O-$(CH_2)_{12}$-$N^+(CH_3)_3$-8%	1.0±0.3
	PAA-C(O)O-$(CH_2)_{12}$-$N^+(CH_3)_3$-14%	1.0±0.4		PAA-C(O)O-$(CH_2)_{12}$-$N^+(CH_3)_3$-14%	0.8±0.3

References:

1. Van Krevelen, D.W., Te Nijenhuis, K. Properties of Polymers. Their Correlation with Chemical Structure; their Numerical Estimation and Prediction from Additive Group Contributions. Elsevier, 2009.
2. Cunico, L.P., Acosta, M.C., Turner, C. Experimental measurements and modelling of curcumin solubility in CO2-expanded ethanol. J. Supercrit. Fluids 2017, 130, 381–388.
3. Subrahmanyam, R., Gurikov, P., Dieringer, P., Sun, M., Smirnova, I. On the Road to Biopolymer Aerogels—Dealing with the Solvent. Gels 2015, 1, 291-313.
4. Guan, J., Liu, Q., Zhang, X., Zhang, Y., Chokshi, R., Wu, H., Mao, S. Alginate as a potential diphase solid dispersion carrier with enhanced drug dissolution and improved storage stability. Eur. J. Pharm. Sci. 2018, 114, 346–355.
